# Identification of Functional Cellular Markers Related to Human Health, Frailty and Chronological Age

**DOI:** 10.1111/acel.70153

**Published:** 2025-07-01

**Authors:** Chloé Brodeau, Camille Joly, Anaïs Chekroun, Jean Nakhle, Vincent Blase, Nicolas Espagnolle, Cédric Dray, Armelle Yart, Valérie Planat, Margot Tertrais, Julien Fassy, Sophie Guyonnet, Wan‐Hsuan Lu, Philipe de Souto Barreto, Olivier Teste, Marie Tremblay‐Franco, Kamaryn T. Tanner, Alan A. Cohen, Audrey Carriere, Louis Casteilla, Isabelle Ader, IHU HealthAge/Open Science group

**Affiliations:** ^1^ RESTORE Research Center Université de Toulouse. INSERM 1301, CNRS 5070, EFS, ENVT Toulouse France; ^2^ Université de Toulouse, UT2J, IRIT, (CNRS/UMR 5505) Toulouse France; ^3^ Institut Hospitalo‐Universitaire HealthAge IHU HealthAge Toulouse France; ^4^ Université Côte d'Azur, UMR CNRS 7275 Inserm 1323, IPMC Valbonne France; ^5^ Gérontopole of Toulouse, Institute of Aging Toulouse University Hospital (CHU) Toulouse France; ^6^ CERPOP UMR 1295 Université de Toulouse, INSERM, UPS Toulouse France; ^7^ Toxalim (Research Center in Food Toxicology) Toulouse University, INRAE, ENVT, INP‐Purpan, UPS Toulouse France; ^8^ Department of Environmental Health Sciences, Butler Columbia Aging Center, Mailman School of Public Health Columbia University New York New York USA

**Keywords:** cellular aging, dermal fibroblast, extracellular matrix, healthy aging, intrinsic capacity, metabolism

## Abstract

Aging leads to a decline in physiological reserves, an increase in age‐related diseases, reduced functional ability and a shortened healthspan. While molecular markers of chronological aging exist, their link to general health and intrinsic capacity (IC), a composite measure of physical and mental capacities, remains unclear. This study integrates the WHO's Healthy Aging framework with geroscience to explore fibroblasts as indicators of health. We assessed primary skin fibroblasts from 133 individuals aged 20–96, evaluating their ability to maintain tissue structure, modulate immune responses and regulate metabolism (SIM functions). By combining functional and molecular analyses, we investigated the relationship between fibroblast performance, chronological age and IC. Our results demonstrate that fibroblast SIM functions are modified with stressors and age, correlating with IC rather than just chronological age. Notably, fibroblasts from pre‐frail and frail individuals exhibited reduced mitochondrial respiration and lower extracellular periostin levels, with periostin being able to capture IC status, irrespective of age and sex, reflecting a cellular ‘health memory’. The SIM paradigm provides a complementary framework to the established hallmarks of aging, advancing our understanding of how cellular aging impacts functional decline. These findings suggest that fibroblast‐derived markers could serve as indicators of frailty and reduced IC, enabling early detection of individuals at risk for health deterioration and laying the foundation for early identification of functional decline.

## Graphical abstract

Human skin fibroblasts in vitro preserve the memory of age and health.

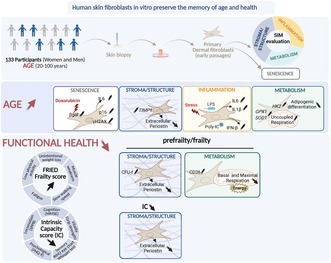

## 1 Introduction

Aging is a physiological process often characterized by declining function over time and is the primary risk factor for many age‐related diseases, impacting health and survival (Niccoli and Partridge 2012). The World Health Organization (WHO) identifies five key functional domains to track health during aging, and frailty—a clinical syndrome marked by reduced strength, endurance, and physiological functions increasing an individual's vulnerability to adverse health outcomes—serves as a key sign of unhealthy aging and a precursor of dependency (Beard et al. 2016; Fried et al. 2001). Intrinsic capacity (IC), defined as the composite of all physical and mental capacities of an individual, is a function‐centered health index based on individual attributes contributing to healthy aging (Beard et al. 2019; Gonzalez‐Bautista and Beard 2023). It is defined as the composite of all physical and mental capacities of an individual and is now recognized as a more reliable, measurable and predictive health indicator than chronological aging. These clinical advances in measuring functional health have been paralleled by notable progress in comprehending the intricate and dynamic cellular changes underlying biological aging. While several cellular/molecular hallmarks have been recognized as pivotal drivers of the chronological aging process (Tartiere et al. 2024), a gap persists in linking cellular changes to individual healthy aging phenotypes such as IC and frailty. The emerging field of geroscience proposes that aging biology is the main driver of chronic disease susceptibility and that targeting it will slow the appearance and progression of age‐related diseases and disabilities (Kennedy et al. 2014). Nevertheless, recent studies have highlighted a discrepancy between chronological aging and molecular markers such as epigenetic clocks (Kabacik et al. 2022), whose underlying mechanisms are often unclear, highlighting the need to identify more reliable markers in the context of general health, rather than age. To overcome the limitations of such strategies, we recently introduced a ‘gerophysiological’ approach, focusing on the complex relationship between physiology, aging, and systemic functional outcomes to assess healthy longevity (Kemoun et al. 2022). While extensive research has been conducted on the discovery of molecular markers associated with chronological aging, the mechanisms leading to age‐related cellular dysfunctions, and the functional phenotypes (intrinsic capacity, frailty, etc.) associated with human healthspan, very few studies connect these three concepts.

To fill the gap between cellular aging processes and individual healthy aging phenotypes, fibroblasts are one of the best candidate cell types to conduct such a study, as they exert a multifunctional role in preserving tissue architecture and function (Plikus et al. 2021), which progressively declines during aging.

Skin behaves as a crucial physical barrier separating internal organs from the external environment, enduring constant exposure to external stresses. Among its constituents, skin fibroblasts play a pivotal role as the primary component of the dermal layer. Beyond their foundational contribution to skin architecture, dermal fibroblasts actively engage in metabolic regulation (Zhao et al. 2019), immune (Haniffa et al. 2007) and inflammatory responses (Sorrell and Caplan 2009), wound healing, and intercellular communication within both the skin ecosystem and the extrinsic skin compartment (Werner et al. 2007). Their relatively low proliferation rate leads to the accumulation of age‐related damage (Tigges et al. 2014), and studies on non‐human models have revealed a correlation between the stress resistance of isolated skin fibroblasts and life expectancy across species (Harper et al. 2007). Moreover, their accessibility via superficial skin biopsies renders them invaluable for investigating human aging, given their distinctive attributes and potential to uncover age and functional decline‐related biomarkers. Numerous multi‐omics studies have documented proteomic, epigenetic and transcriptomic alterations in human skin fibroblasts with age (Solé‐Boldo et al. 2020; Tsitsipatis et al. 2023; Sturm et al. 2022). Notably, RNAseq profiles have allowed the prediction of chronological age (Fleischer et al. 2018) and the biophysical and biomolecular characteristics of human skin fibroblasts have enabled the quantification of cellular aging (Phillip et al. 2017); however, these studies lack critical information regarding the general health state of the individuals. While certain blood biomarkers have been linked to frailty syndrome in older individuals (Sepúlveda et al. 2022), no definitive biomarker‐based diagnostic exists. This would allow for the implementation of suitable preventive or intervention strategies to reduce the likelihood or severity of frailty. Currently, no cellular study has been conducted to determine whether biomarkers of aging are associated with intrinsic capacity regardless of chronological age. Establishing a correlation between biological markers and individual Intrinsic Capacity could enhance our understanding of mechanisms underlying Intrinsic decline and provide targets for risk identification and early interventions. In this study, we propose an integrated characterization of three key pillars of aging—metabolism, inflammation and senescence—combined stroma/structure variables and with a functional assessment of fibroblasts. To this end, we analyzed fibroblasts obtained from skin biopsies of 133 male and female volunteers from the INSPIRE human translational cohort (INSPIRE‐T cohort) (Ader et al. 2021), aged 20–96 years, encompassing a range of frailty statuses (robust, pre‐frail, frail). The resulting data were effectively integrated using multivariate methods, such as homeostatic dysregulation (Cohen et al. 2021), which reflects the inability to maintain internal stability in response to stress or perturbations, with the Mahalanobis distance—a multivariate statistical measure that quantifies how much an individual's physiological profile deviates from a normative reference population**—**being applied at the cellular level for the first time.

The marginal contribution of each parameter pinpointed the relevance of stroma/structure (S) and metabolism (M) related markers specifically associated with aging, frailty syndrome and intrinsic capacity. This model shows potential for further research aimed at unraveling the mechanisms governing intrinsic capacity and leveraging cellular functional assessments to ultimately extend human healthspan.

## 2 Results

### 2.1 Human Skin Fibroblasts Retain Aging Signatures In Vitro

**FIGURE 1:**
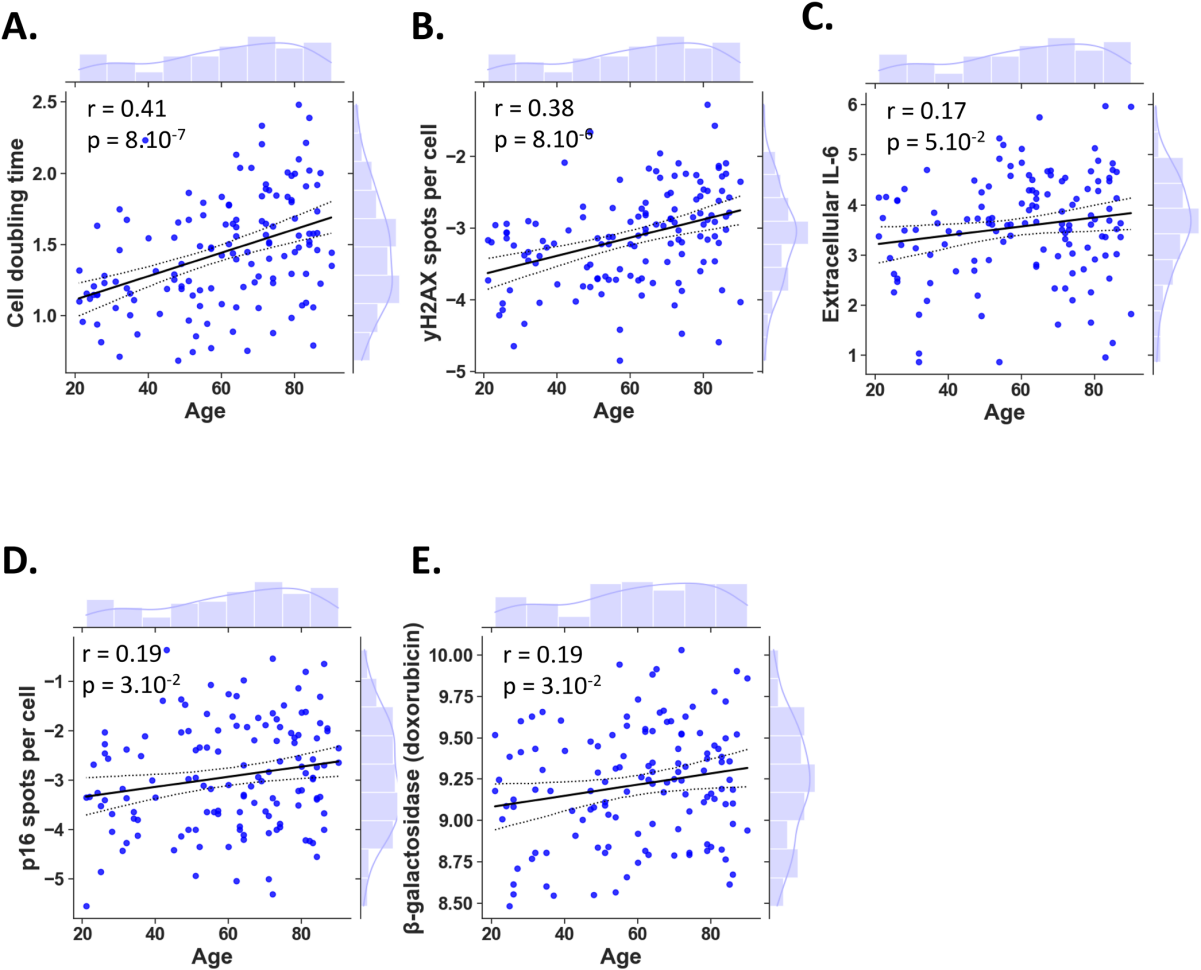
Cellular characteristics of human skin fibroblasts that are significantly correlated with chronological age. Linear regression with marginal distribution represents cell parameters as a function of chronological age. Correlations between age and cell doubling time (days) (A), number of γH2AX foci per cell (B), extracellular IL‐6 concentration (pg/mL/10^5^ cells) (C) and number of p16 spots per cell (D) are shown. The association of senescence associated‐β‐galactosidase (MFI) (E) with age after doxorubicin challenge is shown. The black line represents the regression line, and the dashed line shows the 95% confidence of the fit. Histograms depict the marginal distribution of the respective variable. r and *p*‐value represent the Pearson correlation coefficient and the associated *p*‐value for each measured parameter with age. A *p*‐value < 0.05 was considered significant (A–E).

The first step of our study was to test whether human skin fibroblasts maintained characteristics of aging when cultured in vitro. To do so, we investigated various markers classically associated with cellular aging (López‐Otín et al. 2023). As expected (Rorteau et al. 2022), we observed a positive Pearson correlation between fibroblast doubling time and age, suggesting that the growth of skin fibroblasts was decreased with aging (Figure 1A). Positive Pearson correlations were also observed between age and DNA damage markers, such as the number of γ‐H2AX foci per nucleus (Figure 1B), as well as the inflammatory marker extracellular IL‐6 (Figure 1C) and the number of p16 spots per cell, a marker of cellular senescence (Figure 1D). Of note, the number of γ‐H2AX foci per cell and IL‐6 production and the number of p16 spots per cell were significantly linearly associated (Figure S7A). We had also tested other known markers of cellular senescence such as morphological characteristics like nuclear area, cell size, and cell granularity, as well as Senescence‐Associated β‐Galactosidase (SA‐β‐Gal) activity. None of these markers exhibited a significant increase with age (Figure S2A–D).

### 2.2 Age‐Related Differences in Fibroblasts' Stress Response to Challenges

To better understand how aging impacts cellular resilience, we assessed fibroblasts' responses to stressors that simulate life challenges. Such challenges provide insights into age‐related changes in stress adaptability and senescence induction, which are not always evident under baseline conditions. We used doxorubicin, a chemotherapy drug and potent DNA‐damaging agent, as an inducer of senescence, aiming to determine putative age‐dependent differences in the responses of the donors. Doxorubicin challenge induced a significant increase with age of senescent markers such as SA‐β‐Gal activity with age (Figure 1E). However, none of the other known markers of cellular senescence, such as phosphorylated histone H2AX (γ‐H2AX) foci (Figure S3A), the number of p16 nuclear spots per cell (Figure S3B), morphological characteristics like nuclear area (Figure S3C), cell granularity (Figure S3D) and cell size (Figure S3E) exhibited a significant increase with age.

Taken together, our findings indicate that fibroblasts from the INSPIRE‐T **cohort** exhibit molecular markers associated with chronological age, including cell doubling time and certain markers of senescence such as p16, γ‐H2AX and the pro‐inflammatory cytokine IL‐6.

### 2.3 Simultaneous Assessment of Skin Fibroblast Functional Markers Associated With Age‐Related Decline

**FIGURE 2:**
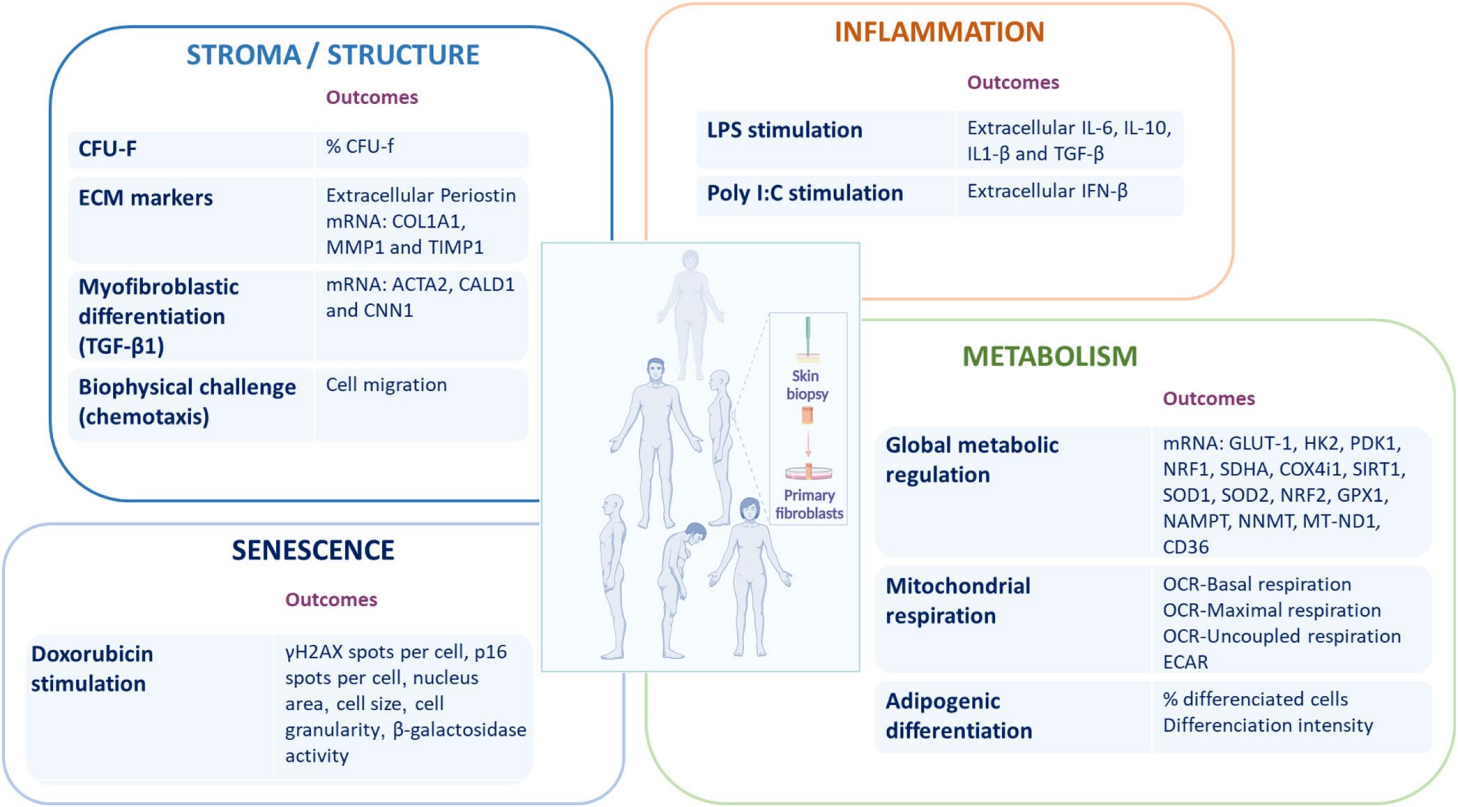
Overview of the study design using dermal fibroblasts from 133 donors selected from the INSPIRE T cohort (ages 20–96 years). Cell characteristics were examined on skin fibroblasts according to the three transverse and interconnected functions—tissue Structuration, Inflammation and the ability to regulate Metabolism (SIM) and senescence. The outcomes measured results per experiments are listed.

To provide a more precise and holistic overview of aging and age‐related decline, and in line with our previous publication (Kemoun et al. 2022), we conducted a detailed integrated approach focusing on metabolism, inflammation, senescence, and stroma/structure using a set of assays to assess the function of dermal fibroblasts from the **cohort of 133 donors** exposed to significant stress (Figure 2). These assays were selected for their ability to identify key molecular markers related to metabolism, inflammation, senescence and stroma/structure and to reflect cellular responses to stress, tissue maintenance, repair and overall health.

The stroma/structure assays focused on fibroblast colony formation (CFU‐F), extracellular matrix (ECM) marker and remodeling enzymes expression (collagen type 1a (*COL1A1*) mRNA, matrix metalloproteinase 1 (*MMP1*) mRNA, tissue inhibitor of metalloproteinase 1 (*TIMP1*) mRNA and Periostin extracellular levels), and myofibroblastic differentiation via TGF‐β1 stimulation. Additionally, cell migration in response to biophysical challenges was assessed. These outcomes informed us about fibroblast proliferative potential, ECM remodeling, and their ability to respond to mechanical stress, reflecting tissue repair capacity.

The inflammation domain was investigated through responses to lipopolysaccharide (LPS) and Poly I:C stimulation, measuring extracellular pro‐inflammatory cytokine release (IL‐6, IL‐10, IL1‐β, IFN‐β and TGF‐β). These assays help us understand the fibroblast's role in immune responses and its potential to contribute to chronic inflammation, a known driver of aging and functional decline.

Finally, metabolic assays assessed global metabolic regulation (mRNA expression of key metabolic genes like glucose transporter 1 (*GLUT‐1*), Hexokinase 2 (*HK2*), pyruvate dehydrogenase kinase 1 (*PDK1*), nuclear respiratory factor 1 and 2 (*NRF1* and *NRF2*), succinate dehydrogenase A (*SDHA*), cytochrome c oxidase subunit 4 isoform 1 (*COX4i1*), sirtuin 1 (*SIRT1*), superoxide dismutase 1 and 2 (*SOD1*, *SOD2*), glutathione peroxydase 1 (*GPX1*), nicotinamide phosphoribosyltransferase (*NAMPT*), nicotinamide N‐methyltransferase (NNMT) and CD36), mitochondrial respiration, and adipogenic differentiation. These measurements give insight into how fibroblasts manage energy, oxidative stress and their capacity for differentiation, which is important for cellular function and adaptation to stress.

Finally, doxorubicin‐induced senescence was explored through γH2AX spots per cell, p16 spots per cell, nucleus area, cell size, cell granularity and β‐galactosidase activity.

Together, these assays provide a multidimensional view of fibroblast health and behavior, enabling us to capture critical aspects of their role in maintaining skin integrity and overall tissue homeostasis across different stages of aging.

### 2.4 Association of Homeostatic Dysregulation With Chronological Age

**FIGURE 3:**
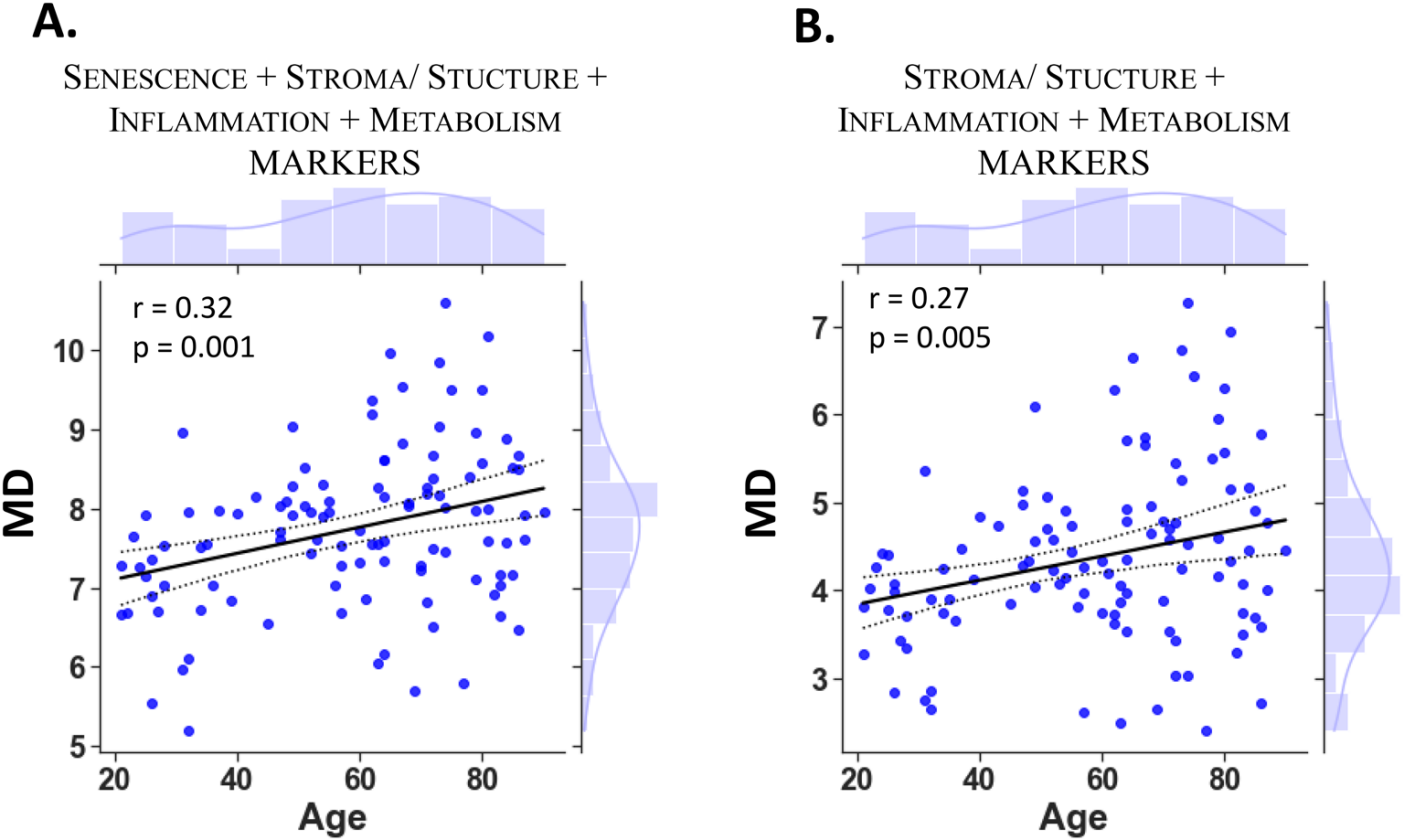
Association of homeostatic dysregulation with chronological age. Scatterplot of homeostatic dysregulation (MD) versus age for *n* = 108 donors with complete data on 31 cellular markers representing: (A) stroma/structure (% CFU‐F, extracellular Periostin, cell migration basal/residual chemoattractant), inflammation (extracellular IL1‐β basal/LPS residual, extracellular IL‐6 basal/LPS residual, extracellular IL‐10 basal/LPS residual, extracellular TGF‐β basal/LPS residual, extracellular IFN‐β basal/Poly I:C residual), metabolism (% differentiated adipocytes, differentiation intensity, OCR‐basal respiration, OCR‐uncoupled respiration and basal ECAR) and senescence (cell size basal/doxorubicin residual, beta galactosidase basal/doxorubicin residual, number of yH2AX spots per cell basal/doxorubicin residual, number of p16 spots per cell basal/doxorubicin residual, nucleus area basal/doxorubicin residual, cell granularity basal/doxorubicin residual) and (B) with SIM markers without senescence. The black line represents the regression line and the dashed lines show the 95% confidence of the fit. Histograms depict the marginal distribution of the respective variable. *r* and *p*‐value represent the Pearson correlation coefficient, and the associated *p*‐value for each measured parameter with age. A *p*‐value < 0.05 was considered significant.

One of the key objectives of our work was to identify signatures associated with chronological age and functional health through an integrative study. Multiparametric analysis using a set of cellular markers has become a promising approach for elucidating the complex mechanisms of aging. Mahalanobis distance is a statistical measure of homeostatic dysregulation and quantifies how different an individual's biomarker levels are from the norm (Cohen et al. 2013). It accounts for correlations between variables, allowing for quantification of a global systemic dysregulation when some variables are highly correlated. High distance indicates high deviation from the population average and is associated with numerous adverse health outcomes (Li et al. 2015; Flores‐Guerrero et al. 2021; Belsky et al. 2018). For this study, we calculated homeostatic dysregulation as the Mahalanobis distance of the largest set of cellular markers feasible across the maximum donor pool. Thus, our analysis was based on 31 cellular markers from 108 donors (Table S2). Using this subset, we investigated the association between homeostatic dysregulation and individuals' age (Figure 3). Our results indicated that the set of 31 Stroma‐Structure/Inflammation/Metabolism and Senescence related markers revealed a homeostatic dysregulation significantly associated with chronological age (Figure 3A). These findings underscore the intricate relationship between cellular markers spanning various biological domains and the aging process, as evidenced by the significant association between homeostatic dysregulation and age. Strikingly, a similar positive association was observed when focusing solely on the homeostatic dysregulation of Stroma‐structure/Inflammation and Metabolism cellular markers, excluding senescence markers (Figure 3B). Altogether, these results show that this integrative SIM approach—comprising Stroma/Structure (S), Inflammation (I) and Metabolism (M)—can be leveraged to predict chronological aging.

### 2.5 Key Molecular Factors Involved in Skin Structure Organization Are Associated With Chronological Aging

**FIGURE 4:**
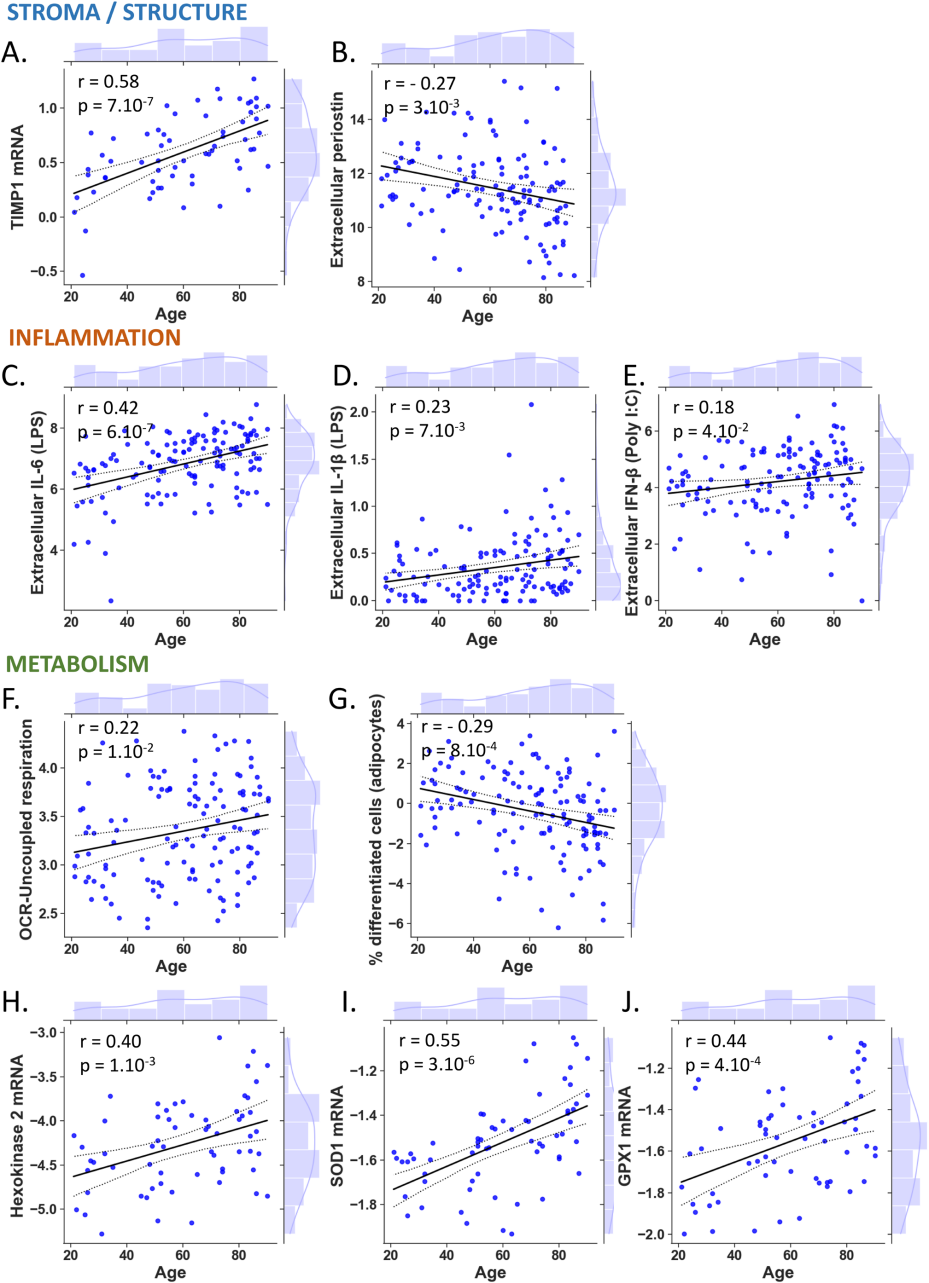
The chronological age affects the regulation of pivotal molecular mediators implicated in the organization of stroma/structure, inflammation and metabolism within skin fibroblasts. Linear regression with marginal distribution represents cell parameters from stroma/structure (A, B), inflammation (C–E) and metabolism (F, G) as a function of chronological age. Correlation between age and *TIMP1* mRNA expression (2^−ΔCt^) (A) and extracellular Periostin logarithmic concentration (pg/mL/10^5^ cells) (B) are shown. Association with age of extracellular IL‐6 (C) and IL1‐β (D) concentration (pg/mL/10^5^ cells) after LPS stimulation (1 μg/mL) and extracellular IFN‐β concentration (pg/mL/10^5^ cells) after Poly I:C stimulation (100 μg/mL) (E) are shown. Correlation between age and uncoupled respiration over basal mitochondrial respiration (%) (F), % differentiated cells (adipocytes) (G) *HEXOKINASE 2* mRNA expression (2^−ΔCt^) (H), mRNA expression (2^−ΔCt^) of *SOD1* (I) and GPX1 (J) are shown. The black line represents the regression line, and the dashed line shows the 95% confidence of the fit. Histograms depict the marginal distribution of the respective variable. *r* and *p*‐value represent the Pearson correlation coefficient and the associated *p*‐value for each measured parameter with age. A *p*‐value < 0.05 was considered significant (A–J).

Aiming to identify markers linked to age, and building on previous findings, we furthered explored potential individual associations between S, I and M markers. The key structural role of fibroblasts is related to their ability to secrete and remodel the extracellular matrix and their ability to differentiate towards myofibroblasts. Thus, we sought to test whether these two functional components were associated with chronological age. Our findings indicated that there was no significant change in *COL1A1* and *MMP1* mRNA levels with age (Figure S2H,I), whereas *TIMP1* mRNA levels exhibited a significant increase (Figure 4A). The evaluation of extracellular Periostin levels, known for its role in ECM organization—particularly in facilitating proper collagen assembly and maintaining homeostasis (Kii et al. 2010; Egbert et al. 2014)—revealed a significant negative correlation with age (Figure 4B). Finally, we evaluated the myofibroblast potency of fibroblasts following stimulation with TGF‐beta. This was achieved by quantifying the level of the of *ACTA2*, *CALD1* and *CNN1* transcripts, known to be expressed during myofibroblast differentiation. The results revealed no significant association between these mRNAs induction and age (Figure S4A–C). These findings highlight that while *COL1A1*, *MMP1* and myofibroblast differentiation markers show no significant age‐related changes, extracellular Periostin and *TIMP1* mRNA levels are correlated with chronological age. This suggests that Periostin and *TIMP1* play important roles in age‐related modifications of the extracellular matrix.

### 2.6 Aging Exacerbates Cytokine Production in Human Skin Fibroblasts

Fibroblasts are recognized for their role in modulating immune system components by secreting signaling factors such as cytokines and growth factors (Plikus et al. 2021). With advancing age, there is a tendency for increased production of pro‐inflammatory cytokines, contributing to the development of chronic, low‐grade inflammation known as inflammaging (Franceschi et al. 2018), and non‐immune cells like fibroblasts have been implicated in this phenomenon through the release of active molecules (Mohan et al. 2011). Our study revealed a significant age‐related increase in extracellular levels of pro‐inflammatory cytokines such as IL‐6 under basal conditions (Figure 1C), while extracellular IL‐1β remained unchanged with age (Figure S5A). Interestingly, extracellular basal levels of IL‐10, TGF‐β, and IFN‐β were consistently undetectable in the supernatants of the majority of donor fibroblasts, regardless of age, under basal conditions (Figure S5B–D). To explore age‐related differences among donors, we investigated how aging influences the inflammatory response of fibroblasts to cellular stressors such as bacterial and viral infections. To simulate these inflammatory challenges, cells were exposed to either the bacterial ligand LPS or the viral PRR ligand poly (I: C), a synthetic analogue of viral dsRNA. Following LPS stimulation, we observed significant positive Pearson correlations between extracellular pro‐inflammatory IL‐6 (Figure 4C) and IL‐1β (Figure 4D) levels and age. Moreover, after LPS stimulation, extracellular IL‐6 and IL‐1β exhibited a significant linear correlation (Figure S7A). However, we found no age‐dependent differences in extracellular IL‐10 and TGF‐β levels (Figure S5E,F). Finally, our results indicated that Poly (I: C) stimulation significantly enhanced the secretion of antiviral IFN‐β in skin fibroblasts, and this increase was positively correlated with age (Figure 4E). In summary, aging enhances the pro‐inflammatory and antiviral responses of skin fibroblasts, with increased secretion of IL‐6, IL‐1β and IFN‐β. These results suggest that fibroblasts play a crucial role in the age‐related amplification of inflammation and immune responses.

### 2.7 Chronological Aging Impairs Metabolic Abilities of Primary Fibroblasts

Numerous studies highlight the importance of energy and redox metabolism in determining cell fate and functions (Zhao et al. 2019; Ghosh‐Choudhary et al. 2020; Santolini et al. 2019). We first investigated mitochondrial metabolic activity by determining oxygen consumption rate (OCR). There was no significant variation of fibroblast OCR basal and maximal respiration or ECAR with age (Figure S6A–C) and the expression of genes regulating mitochondrial metabolism (*NRF1*, *SDHA*, *COX4i1*, *MT‐ND1* and *PDK1*) (Table S3; Figure S6E–I). However, we observed a positive Pearson correlation between age and uncoupled respiration or proton leak (Figure 4F).

The expression of genes related to glucose metabolism including *HK2* which phosphorylates glucose during the first and irreversible step of glycolysis was also significantly increased with age (Figure 4H).

Then, we focused on gene expression of different enzymes involved in the anti‐oxidative response. Pearson correlations showed very significant increase in *SOD1* and *GPx1* mRNA levels with age (Figure 4I,J) whereas we observed no age dependent differences in mRNA *NRF1*, *SIRT1*, *NRF2* and *SOD2* levels (Figure S6C,E,J,K,L). Our findings also revealed significant Pearson correlations between the expression of the *SOD1* gene and the expression of the *GPx1* gene (Figure S7B). Additionally, significant correlations were observed between the expressions of the *SOD1* and *HK2* genes and uncoupled respiration (Figure S7B).

Among their intrinsic abilities, fibroblasts can behave as adipocyte progenitors to store triglycerides and regulate skin homeostasis (Plikus et al. 2021). We challenged their ability to differentiate into adipocytes and observed a negative Pearson correlation showing a linear decrease in the % of differentiated cells with age (Figure 4G).

These results revealed that mitochondrial respiration becomes less efficient with chronological age, associated with increases in glycolytic metabolism and the up‐regulation of the expression of genes involved in antioxidant defense systems.

### 2.8 Extracellular Periostin and Energy Metabolism as Key Markers of Frailty and Intrinsic Capacity in Fibroblasts Independently of Chronological Age and Sex

**FIGURE 5:**
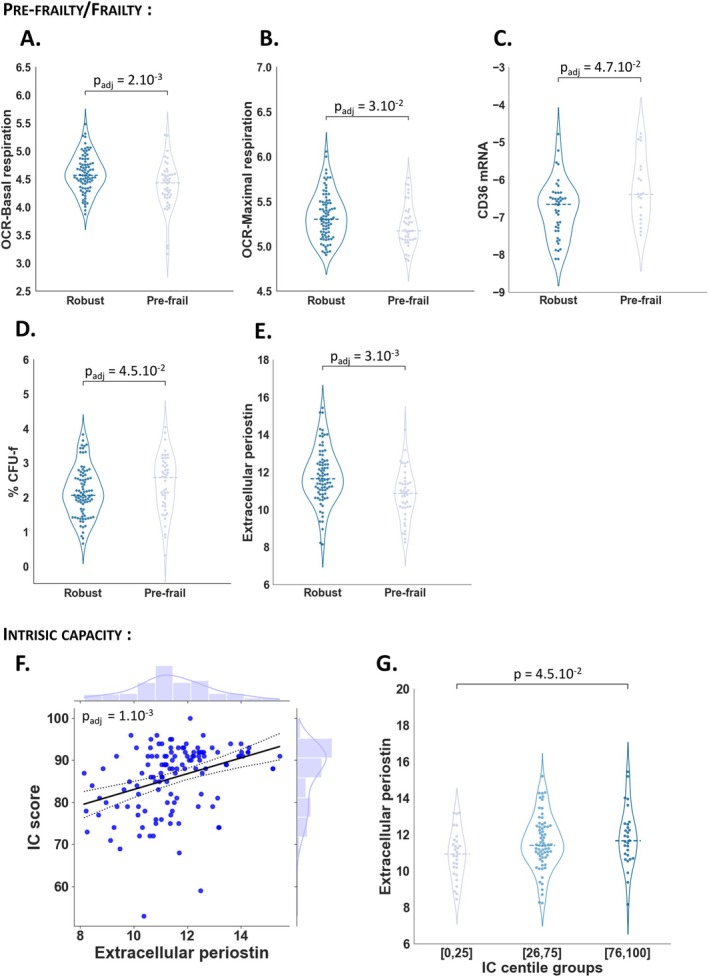
Metabolism and structure human skin fibroblast markers reveal functional decline regardless of chronological age. Violin plots show the distribution of basal and maximal mitochondrial respiration (pmol/min/2.10^4^ cells) (A, B), CD36 mRNA expression (C), % CFU‐f (D) and extracellular Periostin concentration (pg/mL/10^5^ cells) (E) in robust and pre‐frail/frail population. The horizontal line represents the median. Logistic regressions were performed to examine the association between cell parameters and pre‐frailty/frailty status. Cell parameters *p* values ‘*p*_adj_’ are based on logistic regression with adjustment for age and sex (A–E). Linear regression with marginal distribution represents Intrinsic Capacity score (IC) as a function of extracellular Periostin concentration (pg/mL/10^5^cells) and extracellular Periostin *p* value ‘*p*_adj_’ is based on linear regression with adjustment for age and sex. The black line represents the regression line and the dashed line show the 95% confidence of the fit. Histograms depict the marginal distribution of the respective variable (F). Violin plot shows the distribution of extracellular PeriostinN concentration (pg/mL/10^5^ cells) by IC centile group. The horizontal line represents the median. Association between IC centile groups (age and sex specific) and extracellular PERIOSTIN secretion is determined using one‐way ANOVA test (G). A *p*‐value < 0.05 was considered significant (A–G).

Another step in our study was to determine beyond the association with chronological age whether there was an association between the individual variables and the functional health of the participants. Logistic regressions were conducted for each of the cellular markers between fibroblast characteristics and pre‐frailty/frailty status within the study population (Table S3). Among all variables, the senescence and inflammation markers indicated no significant correlation with frailty status, while basal and maximal mitochondrial respiration, *CD36* mRNA expression, percentage of CFU‐f and extracellular Periostin were significantly decreased in fibroblasts from pre‐frail/frail donors compared to the robust group, independently of age and sex (Figure 5A–E). Next, we investigated whether IC—a measure reflecting an individual's combined physical and mental abilities—was associated with age‐related markers in our study (Lu, Rolland, et al. 2023) (Table S3). Only extracellular Periostin was significantly associated with IC score, independently of age and sex as revealed by linear regression analysis, (Figure 5F). Furthermore, by using age‐ and sex‐specific cut‐offs for IC, referenced against centile curves, that stratified participants from the INSPIRE‐T cohort into percentile categories ranging from ≤ 10th to > 90th (Lu, Rolland, et al. 2023), we observed a positive association between centile categories and extracellular Periostin levels (Figure 5G). The concentration of extracellular Periostin was significantly lower in individuals with low IC, classified within the [0.25] percentile range, compared to those in the [76.90] percentile categories. In summary, our findings reveal that extracellular Periostin and basal mitochondrial respiration are key markers associated with frailty and intrinsic capacity (IC), independent of chronological age and sex. Extracellular Periostin, in particular, emerges as a robust indicator of functional health, correlating strongly with IC scores and centile categories.

## 3 Discussion

Our analysis was focused on three interconnected physiological networks: stroma/structure, inflammation and metabolism on human skin fibroblasts from 133 donors aged 20–96 years. With this strategy, we found specific molecular and cellular functional markers not only linked to chronological age but also associated with intrinsic capacity and frailty syndrome.

Our results show that, under standardized in vitro culture conditions, primary skin fibroblasts retain specific features associated with the donor's health status at the time of sampling, regardless of chronological age. These include markers significantly related to frailty and intrinsic capacity, particularly those reflecting tissue stroma/structure (Extracellular Periostin and % CFU‐f), as well as cellular metabolism (including basal and maximal mitochondrial respiration and *CD36* mRNA). These findings underscore the relevance of human fibroblasts as a model system for studying the biological mechanisms underlying aging and healthy longevity. Significant correlations with chronological age were found in 15 out of the 60 measured SIM parameters, far more than expected by chance. This demonstrates the robustness and specificity of the age‐associated markers and underscores the importance of integrating key hallmarks of aging with other cellular functions essential for maintaining tissue functionality and homeostasis. Furthermore, the SIM‐based homeostatic dysregulation, independent of senescent markers, shows a significant association with age. These findings highlight that the age‐related changes in the studied parameters are neither unidirectional nor independent of each other. Instead, they reflect a breakdown in the homeostatic mechanisms that interconnect these parameters. Concerning the individual parameters, we identified two, four and six markers related to the stroma/structure, inflammation and metabolism, respectively. Altogether, these findings largely validate the relevance of the SIM analysis in this context. Our analysis of extracellular matrix (ECM) components identified a decline in Periostin levels with age. The decrease in Periostin in dermal fibroblasts appears to be linked to lower gene expression, as indicated by Egbert et al.'s findings on skin fibroblasts (Egbert et al. 2014). The age‐related reduction in Periostin might compromise cell adhesion and structural integrity while also diminishing the skin's wound‐healing capabilities and other age‐related tissue changes (Cole et al. 2018) because Periostin plays a crucial role in ECM organization, particularly in collagen assembly, and is key to skin repair and wound contraction (Elliott et al. 2012). It also mediates communication between keratinocytes and fibroblasts, influencing keratinocyte function and maintaining skin architecture. Furthermore, Periostin variability suggests its potential as a marker for accelerated aging, independent of chronological age. This marker could be relevant for identifying early signs of functional decline, irrespective of an individual's chronological age. Regarding inflammation, our study found increased levels of IL‐6 in older fibroblasts, which is often linked to oxidative stress and mitochondrial dysfunction (Scheld et al. 2019). Older cells also exhibited a heightened inflammatory response to microbial challenges, producing more IL‐6 and IL‐1β. This could indicate that the fibroblast cells might display a lower capacity to regulate inflammation with aging, making them more vulnerable to chronic inflammatory conditions. Focusing on metabolism, we observed a disruption in energy metabolism with age, characterized by increased uncoupled respiration, and upregulation of certain genes associated with glycolysis (including *HK2*) and the antioxidant response (*GPX1* and *SOD1*). The potential increase in anaerobic glycolysis, uncoupled respiration may trigger a cellular response involving the upregulation of antioxidant enzymes to counteract oxidative damage induced by increased metabolic activity. Our observations show stable mRNA levels of *NRF2* and *NRF1* with aging, suggesting the possible involvement of other transcription factors, such as FOXO, in upregulating antioxidant genes like *SOD1* and *GPX1* (Xing et al. 2018). Our findings on dermal fibroblasts indicate that a dysfunction in mitochondrial metabolism may contribute to increased oxidative stress during aging. However, even though mitochondrial energy metabolism is a primary source of ROS, other molecular factors have also been shown to influence its regulation, including various metabolic pathways (e.g., NADPH oxidases), certain cytokines, and transcription factors like NF‐kB, which increase the expression of antioxidant proteins (Morgan and Liu 2011).

Recent geroscience research suggests that biomarkers of aging can help identify frail individuals, as both aging and related diseases seem to share molecular mechanisms, including inflammation and metabolism (Salvioli et al. 2023). Although intrinsic capacity has not been widely studied in relation to these biomarkers, some research has linked it to inflammation and metabolic markers (Beyene et al. 2024). For instance, Lu WH et al. found connections between plasma biomarkers of inflammation, metabolism, and intrinsic capacity in older adults, but these studies mostly involved blood samples from individuals over 70 (Lu, Guyonnet, et al. 2023). Strikingly, in our study, among all markers associated with chronological age, only reduced Periostin levels and mitochondrial respiration remain associated with health status, whether frailty and/or intrinsic capacity irrespective of age or gender. This finding, supported by evidence linking Periostin to physical and cognitive abilities in the elderly (Sánchez‐Sánchez et al. 2023), reinforces its potential as a marker for intrinsic capacity and frailty. Since Periostin is key to tissue regeneration and wound healing, it likely plays a vital role in maintaining tissue function. The next step will be to decipher whether Periostin may play a causal role in maintaining these functions by exploring its various modes of regulation, which can be quite complex. Whatever the answer, its measure needs to be systematically implemented when healthy aging is addressed.

Another notable observation is the reduced mitochondrial respiration in fibroblasts from pre‐frail and frail individuals. Consistent with the decrease of oxidative capacity in aging (Lesnefsky and Hoppel 2006), this indicates the importance of metabolism in health‐related cellular changes. Furthermore, the age‐related deterioration in both the extracellular matrix and energetic metabolism, highlights the need to better understand their connections to develop strategies for healthier aging. In conclusion, our study highlights the relevance of examining multiple biological factors together through the SIM analysis to gain a holistic view of aging and its impact on health. By integrating markers from domains like senescence, stroma/structure, inflammation, and metabolism, we identified distinct biological signatures tied to both chronological age and functional health. This suggests that aging is not a uniform process but varies across different biological areas, each influencing an individual's health in unique ways. Our approach emphasizes the need for therapeutic strategies focused on preserving metabolic and structural integrity to enhance resilience against age‐related decline, pointing to promising paths for personalized interventions in aging. More specifically, we foresee that the functional assessment of fibroblasts might be a viable strategy in the future to diagnose early functional decline and serve as a rapid outcome for the development of healthspan‐extending therapies.

These findings also carry putative clinical significance. Our data suggest that specific features of dermal fibroblasts—particularly reduced Periostin levels and impaired mitochondrial respiration—may serve as biomarkers of functional decline, including frailty and reduced intrinsic capacity, independently of chronological age and sex. However, translating these findings into clinical practice requires validation in a broader context, followed by the standardization and miniaturization of all steps in the bioassay process to ensure scalability, reproducibility and feasibility in routine healthcare settings. Taken together, these markers offer promising avenues for stratifying individuals at risk and guiding early, personalized interventions aimed at preserving functional health and promoting healthy aging. Nevertheless, it is important to acknowledge some limitations of the present study. While the human fibroblasts used in this study retained some aging signatures in culture, there is still a difference between in vitro and in vivo conditions. Although we took care to conduct our investigations on early passages, cells cultured outside their natural environment may lose certain interactions and signals present in a living organism, limiting the generalization of the results to physiological conditions. Additionally, since the cells used in this study were obtained from skin biopsies, it is likely that we isolated one or more subpopulations of fibroblasts selected by the culture process, which could influence the observed cellular responses and limit the extrapolation of our findings to the entire fibroblast population in vivo. Moreover, as these analyses are exploratory and represent an initial investigation of these associations, no multiple hypothesis correction was applied. We acknowledge this as a limitation here, and underscore the importance of validating our findings in independent studies. In this context, while age, frailty, and IC were all significantly associated with various aspects fibroblast function and age, the associations were far more widespread and clearer for age than for frailty or IC. Globally, we judge that associations with frailty and IC are likely but not certain. Further work will be needed to confirm these findings, though Periostin in particular seemed to have clear and consistent associations. Why age was more consistently associated than IC or frailty is also an important question for future research. Finally, while our analyses were adjusted for age and gender, other factors such as comorbidities, lifestyle, or environmental factors (e.g., sun exposure or pollutants) may not have been adequately accounted for. These elements could affect cellular health and aging markers.

## 4 Methods

### 4.1 Ethics Statement

Ethical and regulatory factors are considered within the INSPIRE‐T cohort study. This study adheres to the principles outlined in the Declaration of Helsinki, which serves as the ethical framework for clinical research involving human subjects. Compliance with these principles is mandatory for all individuals involved in human research. The protocol for the INSPIRE‐T cohort study was reviewed and approved by the French Ethical Committee based in Rennes (CPP Ouest V) in October 2019 and registered on the clinical trials registry website http://clinicaltrials.gov (ID NCT04224038). All participants signed and informed consent enabling data to be used for research purposes.

### 4.2 Clinical Characteristics of Individual Donors in the Skin Fibroblasts Cohort of 133 Donors

Primary human fibroblasts were obtained from skin biopsies collected from 133 individual donors spanning ages 20–96 years in the INSPIRE‐T cohort (Guyonnet et al. 2021). The INSPIRE‐T cohort is a population study of 1000 individuals from Toulouse and surrounding areas (France), including participants aged 20 years and older, with varying levels of functional capacity (from robustness to frailty, and even dependency), followed over 10 years. The characteristics of the 133 selected donors are summarized in Table 1. The mean (standard deviation [SD]) age was 60.5 (19.7) years. Males had a higher Body Mass Index (BMI) and IC score than females.

**TABLE 1:** General characteristics of INSPIRE‐T cohort participants providing skin biopsies for fibroblast isolation in this study.

	Total (*n* = 133)	Male (*n* = 65)	Female (*n* = 68)	*p*
Age (years)	60.5 ± 19.7	61.0 ± 19.8	60.1 ± 19.7	0.810
BMI (kg/m^2^)	25.3 ± 3.8	26.0 ± 3.7	24.6 ± 3.9	0.023
Education				
No diploma or primary	7 (5.3%)	5 (7.7%)	2 (3.0%)	0.142
Secondary	7 (5.3%)	1 (1.5%)	6 (9.0%)	
High school	15 (11.4%)	6 (9.2%)	9 (13.4%)	
University and higher	103 (78.0%)	53 (81.5%)	50 (74.6%)	
Frailty status				
Robust	88 (66.2%)	45 (69.2%)	43 (63.2%)	0.583
Pre‐frail or frail	45 (33.8%)	20 (30.8%)	25 (36.8%)	
IC score (0–100)	85.8 ± 7.9	86.9 ± 7.8	84.8 ± 8.0	0.027

*Note:* Categorical variables were summarized by numbers (percentages) and tested using Fisher's exact test, while continuous variables were presented as mean (standard deviation) and tested using the Mann–Whitney *U* test. Education data were missing for one participant (total sample size, *n* = 132).

Abbreviations: BMI, body mass index; IC, intrinsic capacity.

### 4.3 Skin Biopsies

Skin biopsies from non‐sun‐exposed areas were obtained from 133 volunteers from the INSPIRE human translational cohort (INSPIRE‐T cohort) (Guyonnet et al. 2021). Individuals were recruited according to their chronological age (20–96 years old), sex and frailty status (robust, pre‐frail, frail) (Figure S1). Frailty status was assessed with following criteria: unintentional weight loss, self‐reported exhaustion, weakness (grip strength), slow walking speed, and low physical activity. Individuals with none, one or two and three or more characteristic were classified as robust, prefrail and frail, respectively (Fried et al. 2001). Only two individuals in this cohort were frail; both were grouped with the pre‐frail group for statistical reasons. Intrinsic capacity score was measured as the mean score of the five domains (cognition, locomotion, psychology, vitality and sensory) (vision and hearing) (Lu, Rolland, et al. 2023) (Figure S1). Lu et al. identified several key variables for assessing the IC score: Cognition was measured using the 30‐item Mini‐Mental State Examination (MMSE), with scores ranging from 0 to 30 (higher scores indicate better cognition); Locomotion was evaluated through the Short Physical Performance Battery (SPPB), which includes walking, chair stands, and balance tests, summarized on a scale from 0 to 12 (higher scores are better); Psychological well‐being was assessed using the nine‐item Patient Health Questionnaire for depression (PHQ‐9), where scores range from 0 to 27 (higher scores indicate greater depression severity); Vitality was determined by measuring grip strength in the dominant hand using a hydraulic dynamometer (Jamar; measured in kg); Vision was assessed with visual acuity using the WHO simple eye chart, which scores from 0 to 3 (higher scores reflect better vision); and Hearing was evaluated through the whisper test, scored from 0 to 2 (with higher scores indicating better hearing).

### 4.4 Human Skin Fibroblasts—Cell Culture

Fibroblasts were obtained from 4 mm^3^ skin biopsies, cultured in α‐MEM medium (Life technologies) supplemented with 0.25 units/mL Amphotericin, 100 mg/mL Streptomycin, 100 units/mL Penicillin (ASP) and 20% Fetal Bovine Serum (FBS). Cells were detached by incubating at 37°C for 5 min with trypsin/EDTA solution (Life technologies), numerated using an automated cell counter (Beckman Coulter) Vi‐CELL XR and seeded at constant cell density (2000 cells/cm^2^) until passage 3. Cells were maintained in α‐MEM medium (Life technologies) supplemented with 1% ASP, 1% L‐glutamine and 5% FBS, at 37°C in a humidified incubator with 20% O_2_ and 5% CO_2_. Medium was changed every 2–3 days. All experiments were performed at passage 4.

### 4.5 Cell Biology Assays

Described in Figure 2.

### 4.6 Gene Expression Analysis

Total RNA was isolated using the ReliaPrep RNA Cell Miniprep System (Promega) according to manufacturer recommendations. Total mRNA was reverse‐transcribed into cDNA using the high‐capacity cDNA reverse transcription kit (Life technologies). RT‐qPCR was performed on a Fluidigm BioMarkHD instrument (Fluidigm Corporation, USA) as previously described (Fassy et al. 2021).

#### 4.6.1 cDNA Pre‐Amplification Step

1.25 μL of cDNA were preamplified using the Preamp Master Mix kit (Fluidigm, cat. no. PN 100–5580) according to the manufacturer's instructions: 1 μL of Preamp Master Mix was combined with 0.5 μL of Pooled Delta Gene assay mix (500 nM) and 1.25 μL of cDNA in a 5 μL total volume reaction. Thermal cycling conditions were: 95°C for 2 min followed by 15 cycles of 95°C for 15 s, 60°C for 2 min.

#### 4.6.2 Clean Up Reaction With Exonuclease I

After each preamplification reaction, samples were cleaned up with exonuclease I (New England Biolabs cat. no. PN‐M0293S) treatment according to the manufacturer's instructions. 2 μL of diluted Exo I at 4 U/μL added to each 5‐μL preamplification reaction. Thermal cycle the Exo I reaction using the following conditions: 37°C for 30 min, 80°C for 15 min. Then, samples were diluted 1:20 by adding 93 μL nuclease‐free water for a 100 μL total volume.

Real time qPCR using BiomarkTM HD system. PCR was performed following Gene Expression with the 192.24 IFC Using Delta Gene Assays protocol (PN 100‐7222 C1), using a 10X assays mix and a pre‐sample mix prepared separately. The 10× assays mix was prepared by mixing 0.2 μL of 100 μM each Delta Gene primers (forward and reverse combined), 2 μL 2X Assay Loading Reagent (Fluidigm PN 100‐7611) and 1.8 μL of 1X DNA suspension buffer to a final volume of 4 μL (per reaction).

The pre‐sample mix was prepared by mixing 2 μL of 2X SsoFast EvaGreen Supermix with low ROX (Bio‐Rad PN 172‐5211), 0.2 μL 192.24 Delta Gene Sample Reagent (Fluidigm PN 100–6653) and 1.8 μL pre‐amplified and Exo I treated cDNA to a final volume of 4 μL. Then, 3 μL of 10× assays mix and of pre‐sample mix are transferred into the 192.24 IFC, loaded into the BiomarkTM IFC controller RX and transferred to the BiomarkTM HD apparatus. Thermal cycling conditions were as follows: 50°C for 120 s, 95°C for 600 s followed by 40 cycles of 95°C for 15 s, 60°C for 60 s.

#### 4.6.3 Gene Expression Determination

The ΔCt was obtained by normalizing mean expression values of each gene to the geometric mean of the reference genes, ribosomal protein lateral stalk subunit P0 (RPLP0) and peptidylprolyl isomerase A (PPIA). Gene expression was calculated by the 2^−ΔCT^ method or in fold increase of 2^−ΔCT^ to control cells. Primer sequences are listed in Table S1. These experiments were conducted on fibroblasts from 65 individuals from the INSPIRE‐T cohort. 3 technical replicates were performed for each condition and donor.

### 4.7 Senescence Induction With Doxorubicin

Treated cells were seeded at 1 × 10^4^ cells/cm^2^ in 24 well plates for immunofluorescence staining and in 6 well plates for C12FDG staining, and were incubated for 24 h with 250 nM doxorubicin. Then, cells were carefully washed with PBS before being cultured in standard medium. Control cells were seeded at 1 × 10^3^ cells/cm^2^ to maintain their proliferation for long‐term culture. Medium was changed every 2–3 days for 10 days after treatment. 3 technical replicates were plated for each condition and donor. These experiments were conducted on fibroblasts from 133 individuals from the INSPIRE‐T cohort.

### 4.8 SA‐β‐Gal Activity Measurement by C12‐FDG Staining

10 days after doxorubicin treatment, cells were treated with 100 nM Bafilomycin 1 (Invivogen tlrl‐bafa1) for 2 h then incubated with 20 μM 5‐Dodecanoylaminofluorescein Di‐β‐D‐Galactopyranoside substrate (C12FDG, Abcam ab273642) in the dark for 1 h at 37°C. Cells were then washed with PBS and nuclei were stained with DAPI. C12FDG uptake was quantified by flow cytometry using a MACSQuant (Miltenyi) on the FITC channel. Mean fluorescent intensity (MFI) was evaluated using Kaluza software. 3 technical replicates were performed for each condition and donor.

### 4.9 Immunofluorescence

10 days after doxorubicin treatment, cells were fixed using 4% paraformaldehyde (PFA) at RT for 20 min. Next, cells were permeabilized and blocked using solution (0.2% Triton X‐100, 2% NHS) at RT for 20 min. Then, cells were labeled with mouse anti‐human phospho‐histone H2AX (Ser139) antibody (1:1000, Merk Millipore) for γ‐H2AX DNA damages foci and with rabbit anti‐human p16 antibody (1:270, Abcam) overnight at 4°C. After washing, their respective secondary antibodies were incubated at RT for 2 h: donkey anti‐mouse Al488 secondary antibody (1:400, ThermoFisher) and donkey anti‐rabbit Al555 secondary antibody (1:200, ThermoFisher). Finally, after repeated washing, nuclei were stained with DAPI at RT for 20 min. Images were taken with the high content imaging system Operetta (Perkin Elmer). γ‐H2AX foci number per cell, p16 nuclear spots number per cell and nuclear area were calculated Harmony software (Perkin Elmer). 3 technical replicates for each condition and donor.

### 4.10 Population Doubling Assay

During fibroblasts amplification, cells were seeded at 2000 cells/cm^2^ in T‐175 cell culture flasks. At 90% cell confluence, fibroblasts were harvested and enumerated. Doubling times were calculated using the following formula: DT = ln2 × *t*/(ln C1‐ln C0), where *t* is the culture duration, C1 is the number of cells at the end of the culture, and C0 is the number of seeded cells. Doubling times of fibroblasts at passage 2 were studied. These experiments were conducted on fibroblasts from 133 individuals from the INSPIRE‐T cohort.

### 4.11 Colony Forming Unit (CFU) Assay

Fibroblasts were plated in 96 well plates by creating a range of cells with serial half‐dilutions, where each point of the range is seeded in 12 technical replicates. The medium was changed every 2–3 days. The cultures were kept at 37°C with 5% CO_2_, and at Day 11 of growth, plates were fixed and stained with DAPI (1:10000, Sigma). Images were acquired using the Operetta High Content Analysis system (Revvity) with 10× NA 0.4; 15 fields of view were captured per well. Harmony analysis software (Revvity) was used to obtain automatically Colony‐formation assay involved using Poisson distribution statistics by determining the number of wells with no clonogenic growth at Day 11. These experiments were conducted on fibroblasts from 133 individuals from the INSPIRE‐T cohort.

### 4.12 Cell Migration Assay

Fibroblast migration was performed using the IncuCyte S3 Live‐Cell Analysis System (v20192.3.7219.27517‐1, 2019B Rev2 GUI, Essenbioscience). Cells were seeded at 1500 cells/well density in α‐MEM medium (Life technologies) supplemented with 1% ASP and 0.1% FBS in a 96‐well plate (top). 200 μL of α‐MEM medium (Life technologies) supplemented with 1% ASP and 10% FBS was added in wells of the reservoir plate (bottom). 3 technical replicates were plated for each condition and donor. Fibroblast migration across the membrane surface through the pores was automatically quantified for 24 h. The total count of fibroblast per well (bottom) normalized to the initial count (top) was calculated without (spontaneous migration) and with chemoattractant (cell migration under chemoattractant) via the IncuCyte S3 software (Essenbioscience). These experiments were conducted on fibroblasts from 133 individuals from the INSPIRE‐T cohort.

### 4.13 Myofibroblastic Differentiation

Cells were seeded at 1.5 × 10^4^ cells/well in 24 well plates and were cultured in standard conditions for 96 h (time to reach cell confluence). Myofibroblastic differentiation was induced with 2 ng/mL TGF‐β1 (Miltenyi) for 10 days. Medium was changed every 2–3 days. RT‐qPCR was performed for the smooth muscle actin (*α‐SMA*), caldesmon (*CALD1*), calponin (*CNN1*), collagen type 1a (*COL1A1*) matrix metalloproteinase 1 (MMP1), tissue inhibitor of metalloproteinase 1 (*TIMP1*) and *Periostin* gene expression. Untreated cells were analyzed when cells reached confluency. 3 technical replicates were plated for each condition and donor. These experiments were conducted on fibroblasts from 65 individuals from INSPIRE‐T cohort. Primer sequences are listed in Table S1.

### 4.14 Adipogenic Differentiation

Cells were seeded at 1.25 × 10^4^ cells/well in 24 well plates and were cultured in standard conditions for 96 h (time to reach cell confluence). Adipogenic differentiation was induced with (1 μM dexamethasone, 60 μM indomethacine and 500 μM IBMX) (Sigma Aldrich) for 14 days. Medium was changed every 2–3 days. Control cells were seeded in standard medium at 1 × 103 cells/cm^2^ to maintain their proliferation for long term culture. 3 technical replicates were plated for each condition and donor. Fibroblasts were fixed, nuclei were marked with DAPI (1:10000, Sigma), and lipid droplets were marked with Bodipy 493/503 (1:500). Images were acquired using the Operetta High Content Analysis system (Revvity) with 20× Air/0.45 NA; 99 fields of view were captured per well. DAPI and Bodipy were excited with the 360–400 and 460–490 nm excitation filters, respectively. Nuclei and cells were segmented with ‘Find nuclei’ and ‘Find cytoplasm’ building blocks. Bodipy+/total cells were quantified using the Harmony Analysis Software (Revvity). These experiments were conducted on fibroblasts from 133 individuals from the INSPIRE‐T cohort.

### 4.15 LPS and Poly IC Stimulation

Dermal fibroblasts (control and treated conditions) were seeded at 5 × 10^4^ cells/cm^2^ in 24 well plates and treated by LPS (1 μg/mL) or Poly IC (100 μg/mL) for 1 h. Untreated fibroblasts were used as control condition. Then, cells were carefully washed four times with PBS before being maintain in standard medium for 48 h. 3 technical replicates were plated for each condition and donor. Conditioned cell culture supernatants were collected and used for cytokine and chemokine profiling by ELLA method. These experiments were conducted on fibroblasts from 133 individuals from INSPIRE‐T cohort.

### 4.16 Simple Plex Assays on Ella

Supernatants were stored at −80°C. IL‐6, IL‐10, IL1‐β and TGF‐β concentrations were measured after LPS stimulation and the IFN‐β concentration was measured after Poly IC stimulation. All of these cytokines have been quantified in control supernatants. Periostin concentration was also measured in control supernatant. Proteins were quantified by disposable microfluidic single and multianalyte SimplePlex cartridge using the fully automated immunoassay platform, Ella (ProteinSimple/Bio‐techne, San Jose, CA). The supernatant samples were thawed on ice and diluted in sample diluent (SD 13) if necessary and loaded into cartridges with relevant high and low control concentrates. Five panels were used: IL‐10 and IL1‐β as pure; TGF‐β as pure; IFN‐β at a dilution of 1:2, IL‐6 at a dilution of 1:4 and Periostin at a dilution of 1:2. Each protein channel contains 3 analyte‐specific glass nanoreactors, which allows for each supernatant sample to be run in triplicates for target protein samples (3 technical replicates). Cartridges include a built‐in lot‐specific standard curve for defined supernatant protein. All steps in the procedure were run automatically by the instrument with no user activity. The obtained data were displayed as pg/mL and automatically calculated by the internal instrument software. Results were normalized to cell number measured during supernatant collection.

### 4.17 Metabolic Flux Analysis

Seahorse XFe96 cell culture microplates were coated overnight with poly‐D‐lysine (0.1 mg/mL). The following day, the wells were thoroughly washed with PBS and allowed to dry for 1 h. Fibroblasts were seeded at 20,000 cells per well in complete α‐MEM medium (Life technologies) and incubated overnight at 37°C in 5% CO_2_. 6 technical replicates were plated for each donor. Sensor cartridges were hydrated overnight in Seahorse XF calibrant at 37°C without CO_2_ supplementation. On the day of the assay, fibroblasts were carefully washed with PBS, placed in XF DMEM supplemented with glucose (10 mM), glutamine (2 mM) and pyruvate (1 mM) then incubated for 1 h at 37°C without CO_2_ supplementation. After Seahorse XFe96 Analyzer calibration, the Oxygen Consumption Rate (OCR) was measured at the basal level, then after the sequential injection of oligomycin (2.5 μM), FCCP (6 μM) and rotenone (2.5 μM) + antimycin A (2.5 μM). 3 cycles were performed at each step, consisting of 3 min of mix followed by 3 min of measurement. At the end of the experiment, the nuclei were stained with DAPI, and the number of nuclei per well was determined using the Operetta High Content Imaging System (Perkin Elmer) and used to normalize the raw OCR data. This protocol provides information on basal respiration (basal OCR), uncoupled respiration (as a percentage of basal respiration), maximal respiration (maximal OCR) and extracellular acidification rate (basal ECAR). Cell density, plate coating, as well as oligomycin and FCCP concentrations were determined by a dedicated pilot experiment. These experiments were conducted on fibroblasts from 133 individuals from the INSPIRE‐T cohort.

### 4.18 Statistical Analysis

Statistical analysis was performed using both Python and Jupyter notebook, and GraphPad Prism version 10 for Windows (GraphPad Software, USA). The Python package pandas was used for data manipulation. Packages matplotlib and seaborn were used for plotting graphs, and the statsmodels module was used for statistics. Associations between each cell parameter and chronological age were assessed using linear regression with the cell parameter as the outcome. Pearson correlation coefficients were also calculated. Associations with intrinsic capacity (IC) were evaluated using linear regression with IC as the outcome; these models were adjusted for age and sex. Associations with frailty status were evaluated using logistic regression with frail/pre‐frail versus non‐frail as the outcome, adjusting for age and sex. Associations between IC centile and cell parameters were determined using a one‐way ANOVA test.

Depending on their distribution and characteristics, variables were transformed using either a natural logarithm (ln), a shifted logarithm (ln(*x* + 1)) to account for zero values, or a reciprocal transformation (1/*x*) to satisfy the assumptions of homoscedasticity and normality of residuals, as assessed by the Breusch‐Pagan test and residual plots, before performing linear regression analyses. For associations with age and frailty status, all variables were log‐transformed, except for extracellular IL‐1β, IL‐1β (LPS), IL‐10, IL‐10 (LPS), TGF‐β, TGF‐β (LPS), INF‐β and INF‐β (Poly I: C), which were transformed using a shifted logarithm [ln(*x* + 1)]. For associations with IC, all variables were log‐transformed, except for β‐galactosidase, CNN1, and MMP1 mRNA fold increase (TGF‐β), and extracellular IFN‐β and TGF‐β (raw values); extracellular IL‐1β, IL‐1β (LPS), IL‐10, IL‐10 (LPS), TGF‐β (LPS) and IFN‐β (Poly I: C), which were transformed using a shifted logarithm [ln(*x* + 1)]; and % differentiated adipocytes, intensity of adipose differentiation, OCR‐Basal and maximal respiration, which were transformed using a reciprocal transformation (1/*x*).

While no formal adjustment for multiple comparisons was used, we carefully considered the risk of false positives in the analysis and presentation. From an ensemble of results, the null expectation is 5% false positives at alpha = 0.05. We would not interpret results as meaningful unless the number of positives far exceeds this. By presenting both positive and negative findings and considering them as an ensemble, we avoid the risk of cherry picking a few false positive results. Some positive results may nonetheless be false positives, but it is impossible to distinguish precisely which ones, and we ensure that our broad conclusions do not hinge on the result of any single test.

The package sklearn decomposition module was used respectively for dimensionality reduction analysis. Using GraphPad Prism, the Mann–Whitney test for two independent samples was used to estimate the difference between two groups.

Mahalanobis distance, a measure of homeostatic dysregulation (Cohen et al. 2013), calculates how unusual a biomarker profile is relative to a population average. It is given by the equation:

$$D_{M}\left(x\right)=\sqrt{{\left(x-\mu \right)}^{T}S^{-1}\left(x-\mu \right)}$$

where *x* is a vector of biological parameter values for a given individual at a given time, μ is the vector of mean values for those same parameters, and *S* is the correlation matrix among those parameters. Both *x* and *S* are calculated from a reference population—often the entire study population—and it is with respect to the multivariate mean of this population that distance is measured. It has previously been widely applied epidemiologically and in various species using standard clinical blood biomarkers (Milot et al. 2014) (Mian et al. 2022) (Senior et al. 2022). High *D*_*M*_ indicates high deviation from the mean and is broadly associated with worse health outcomes and adverse profiles on determinants of health (Li et al. 2015; Flores‐Guerrero et al. 2021; Belsky et al. 2018; Milot et al. 2014; Mian et al. 2022; Senior et al. 2022; Chaney and Wiley 2023) it has also been shown to mediate relationships between determinants of health and health outcomes (Jin et al. 2022; Li et al. 2024). This is, to our knowledge, the first application to cell culture variables. *D*_*M*_ was calculated in R v4.3.1 using observations with complete data on 31 features (all except gene expression markers). Features with right‐skewed distributions were log transformed, and all features were scaled to have a mean of 0 and a standard deviation of 1 prior to analysis. For variables with both a basal level and a post‐challenge level, the basal level and the residuals of a linear regression of the post‐challenge level on the basal level were included. The Mahalanobis distance for each individual was log transformed and standardized by dividing by the sample standard deviation.

## Author Contributions

**Chloé Brodeau:** investigation, methodology, visualization, conceptualization, writing – original draft, writing – review and editing. **Camille Joly**, **Anaïs Chekroun** and **Jean Nakhle:** investigation, methodology, visualization, writing – review and editing. **Marie Tremblay‐Franco** and **Vincent Blase:** methodology, writing – review and editing. **Nicolas Espagnolle:** methodology, conceptualization; writing – review and editing. **Cédric Dray** and **Armelle Yart:** writing – review and editing. **Valérie Planat:** methodology, writing – review and editing. **Margot Tertrais** and **Julien Fassy:** investigation, methodology, visualization, writing – review and editing. **Sophie Guyonnet:** methodology, resources, writing – review and editing. **Wan‐Hsuan Lu** and **Philipe de Souto Barreto:** investigation, methodology, writing – review and editing. **Olivier Teste:** methodology, writing – review and editing. **Kamaryn T. Tanner and Alan A. Cohen:** investigation, visualization, conceptualization, writing – review and editing. **Audrey Carriere:** conceptualization, methodology, writing – review and editing. **Louis Casteilla** and **Isabelle Ader:** supervision, conceptualization, methodology, visualization, writing – original draft, writing – review and editing, funding acquisition.

## Conflicts of Interest

The authors declare no conflicts of interest.

## Supporting information


Appendix S1.

**Table S1.** List of primers used for qPCR assays.

**Table S2.** List of the 31 measured cellular parameters used to determine Mahalanobis distance.

**Table S3.** Association of the 60 measured cellular parameters with chronological age or Intrinsic capacity using linear regression and with frailty using logistic regression. Statistical models were applied to assess the relationship between cellular features and clinical variables. Linear regression models were used to evaluate associations between each of the 60 cellular parameters and chronological age or intrinsic capacity (IC), while logistic regression models were used for frailty. For each parameter, the regression coefficient (β), 95% confidence interval (95% CI), and *p*‐value are reported. *r* is the Pearson correlation coefficient. Cellular parameters are categorized into four functional groups: senescence, stroma/structure, metabolism and inflammation. Statistically significant associations (*p* < 0.05) are indicated in bold.

**Figure S1.** Study population. Distribution of individuals in the fibroblast cohort from INSPIRE‐T cohort according to their chronological age range, sex and frailty status (robust, pre‐frail, frail) (A) and to Fried’s criteria (unintentional weight loss, fatigue, weakness, slow walking speed and low physical activity) (B). Distribution of average Intrinsic Capacity domain scores (Cognition, Locomotion, Psychological, Vitality and Sensory) by IC centile groups and sex (male and female), including the number of individuals in each group (C).

**Figure S2.** Stroma and structure characteristics of skin fibroblasts with chronological age. Linear regression with marginal distribution represents cell parameters as a function of chronological age. Correlation between age and nuclear area (μm^2^) (A), cell size (a.u) (B), cell granularity (a.u) (C), β‐galactosidase (doxorubicin) (MFI) (D), fibroblast clonogenicity potential (% CFU‐f) (E), spontaneous cell migration (a.u) (F) and cell migration in response to 10% FBS as chemoattractant (a.u) (G) are shown. Association of COL1A1 and MMP1 mRNA expression (2^−ΔCt^) with age are shown (H–I). The black line represents the regression line and the dashed line show the 95% confidence of the fit. Histograms depict the marginal distribution of the respective variable. *r* and *p*‐value represent the Pearson correlation coefficient, and the associated *p*‐value for each measured parameter with age. A *p*‐value < 0.05 was considered significant (A–I).

**Figure S3.** Fibroblast response to doxorubicin exposure with chronological age. Linear regression with marginal distribution represents cell parameters as a function of age. Association of number of γH2AX foci per cell (A), number of p16 spots per cell (B), nuclear area (μm^2^) (C) and cell granularity (a.u) (D) and cell size (E) with age after doxorubicin challenge are shown. The black line represents the regression line and the dashed line show the 95% confidence of the fit. Histograms depict the marginal distribution of the respective variable. *r* and *p*‐value represent the Pearson correlation coefficient, and the associated *p*‐value for each measured parameter with age. A *p*‐value < 0.05 was considered significant (A–E).

**Figure S4.** Fibroblast ability to differentiate into myofibroblasts and adipocytes with chronological age. Linear regression with marginal distribution represents cell parameters as a function of age. Association of ACTA2 (A), CALD1 (B), CNN1 (C) mRNA expression fold increase to control cells (2^−ΔΔCt^) with age after myofibroblastic differentiation (2 ng/mL of TGF‐β1 for 10 days) are shown. Correlation between age and intensity of adipose differentiation (D) is shown after 14 days of adipose differentiation induction. The black line represents the regression line and the dashed line show the 95% confidence of the fit. Histograms depict the marginal distribution of the respective variable. *r* and *p*‐value represent the Pearson correlation coefficient, and the associated *p*‐value for each measured parameter with age. A *p*‐value < 0.05 was considered significant (A–D).

**Figure S5.** Cytokine production by fibroblasts under basal conditions and in response to inflammatory stress with aging. Linear regression with marginal distribution represents cell parameters as a function of age. Correlation between age with extracellular IL1‐β (A), IL‐10 (B), TGF‐β (C), and IFN‐β (D) concentration (pg/mL/105 cells) are shown. Association of extracellular IL‐10 (E) and TGF‐β (F) concentration (pg/mL/105 cells) with age after LPS stimulation (1 μg/mL) are shown. The black line represents the regression line and the dashed line show the 95% confidence of the fit. Histograms depict the marginal distribution of the respective variable. *r* and *p*‐value represent the Pearson correlation coefficient, and the associated *p*‐value for each measured parameter with age. A *p*‐value < 0.05 was considered significant (A–F).

**Figure S6.** Chronological age‐related variation in metabolic abilities and in the expression of genes regulating metabolism. Linear regression with marginal distribution represents cell parameters as a function of age. Correlation between age with basal (A) and maximal (B) mitochondrial respiration (pmol/min/2.104 cells), ECAR (C) and GLUT‐1 (D), NRF1 (E), SDHA (F), COX4i1 (G), MT‐ND1 (H), PDK1 (I), SIRT1 (J), NRF2 (K) and SOD2 (L) mRNA expression (2^−ΔCt^) are shown. The black line represents the regression line and the dashed line show the 95% confidence of the fit. Histograms depict the marginal distribution of the respective variable. *r* and *p*‐value represent the Pearson correlation coefficient, and the associated *p*‐value for each measured parameter with age. A *p*‐value < 0.05 was considered significant (A–L).

**Figure S7.** Correlation matrix for variables significantly correlated with chronological age. Pearson’s correlation coefficients were calculated to indicate the strength of the relationship between two variables. The color scale represents the value of the correlation. A *p*‐value < 0.05 was considered significant (*p‐value < 0.05, **p‐value < 0.01, ***p ‐value < 0.001 and ****p ‐value < 0.0001), and those > 0.05 were considered nonsignificant (ns). Correlation matrix of 11 parameters measured on fibroblasts from 119 donors (A). Correlation matrix of 15 parameters measured on fibroblasts from 57 donors (B).

## Data Availability

All data are available in the main text or Supporting Information.
