# Supplementary material for: Identification of Functional Cellular Markers Related to Human Health, Frailty and Chronological Age
Source: Aging Cell. 2025 Jul 1;24(9):e70153. doi: 10.1111/acel.70153 (PMC12419852; doi:10.1111/acel.70153)
Supplement: Supplementary file 1 — Appendix S1. [file ACEL-24-e70153-s003.docx]

**AGING CELL AUTHOR CHECKLIST**. ***Authors should submit this checklist together with their manuscript. Please ensure that you have read the Author Guidelines in detail before submission.***

| **Title** | **Identification of functional cellular markers related to human health, frailty and chronological age** | | | | | | |
| --- | --- | --- | --- | --- | --- | --- | --- |
| **Authors** | Ch Chloé Brodeau, Camille Joly, Anaïs Chekroun, Jean Nakhle, Vincent Blase,  Nicolas Espagnolle, Cédric Dray, Armelle Yart, Valérie Planat, Margot Tertrais, Julien Fassy, Sophie Guyonnet , Wan-Hsuan Lu, Philipe de Souto Barreto, Olivier Teste, Marie Tremblay-Franco, Kamaryn T Tanner, Alan A Cohen, Audrey Carriere, Louis Casteilla, Isabelle Ader. | | | | | | |
| **Manuscript Type** | **Research Article** | | | | | | |
| **Total Character Count (including spaces)^1^** | **54287** | | | | | | |
| **Word count of Summary^2^** | **219 words** | | | | | | |
| **Number of papers cited in the References^3^** | **57** | | | | | | |
| **Listing of all Tables (Table1, Table 2 etc)^4^** | **Table 1:** General characteristics of human skin fibroblasts used in this study from participants of the INSPIRE Human Translational Research Cohort.  **Supplementary table 1**. List of primers used for qPCR assays.  **Supplementary table 2.** List of the 31 measured cellular parameters used to determine Mahalanobis distance.  **Supplementary Table 3.** Association of the 60 measured cellular parameters with chronological age or Intrinsic capacity and with frailty. | | | | | | |
|  |  | | | | | | |
|  |  | | | | | | |
| **Figure specifications (please complete one row per figure)^5^**  ***Figure no.*** | Colour  ***(yes/no)*** | Greyscale  ***(yes/no)*** | Black and white  ***(yes/no)*** | Single column (80mm)  ***(yes/no)*** | Double column (180mm)  ***(yes/no)*** | Size of figure at full scale  (mm x mm)  ***(insert details)*** | Smallest font size used in the figure at full scale (minimum 6pt)  ***(insert***  ***details)*** |
| Fig 1 | YES |  |  |  |  |  | Font used is DejaVu Sans. Axis legend font size is 24 pt; tick and axis label font size is 20 pt. |
| Fig 2 | YES |  |  |  |  |  |  |
| Fig 3 | YES |  |  |  |  |  | • X-axis: Font used is *DejaVu Sans*, legend font size 24 pt, label size 22 pt.  • Y-axis: Font used is *Calibri*, legend and label font size 16 pt. |
| Fig 4 | YES |  |  |  |  |  | Font used is DejaVu Sans. Axis legend font size is 24 pt; tick and axis label font size is 20 pt. |
| Fig 5 | YES |  |  |  |  |  | • Panels A–E: Font used is *DejaVu Sans*, Y-axis legend font size 26 pt, label size 22 pt.  • Panel F: Font used is *DejaVu Sans*, X and Y-axis legend font size 24 pt, label size 20 pt.  • Panel G: Font used is *DejaVu Sans*, X and Y-axis legend font size 26 pt, label size 22 pt. |
| Table 1 |  |  | YES |  |  |  |  |
|  |  |  |  |  |  |  |  |

**^1^** The maximum character count allowed is 50,000 (incl. spaces) for Primary Research Papers and Reviews, 10,000 for Short Takes.

**^2^** Summary should not exceed 250 words.

**^3^** Primary Research Papers can contain a maximum of two tables. If more are needed they should replace some of the Figures or can be placed in the Supporting Information.

**^4^** A maximum of 45 references is allowed for Primary Research Papers and 20 references for Short Takes.

**^5^** A Primary Research Paper may contain up to 6 figures and a Short Take up to 2 figures. Authors are encouraged to provide figures in the size they are to appear in the journal and at the specifications given.
