# Supplementary material for: Identification of Functional Cellular Markers Related to Human Health, Frailty and Chronological Age
Source: Aging Cell. 2025 Jul 1;24(9):e70153. doi: 10.1111/acel.70153 (PMC12419852; doi:10.1111/acel.70153)
Supplement: Supplementary file 2 — Table S1. List of primers used for qPCR assays. [file ACEL-24-e70153-s001.pdf]

Gene	Forward 5' primer sequence	Reverse 5' primer sequence
**ACTA2**	CTATGCCTCTGGACGCACAAC	CAGATCCAGACGCATGATGGC
**CALD1**	AGATTCAAAGGCGAAGAGCA	TTCAAGCCAGCAGTTTCCTT
**CD36**	CGGCTGCAGGTCAACCTATT	CACCAATGGTCCCAGTCTCA
**CNN1**	CCAACGACCTGTTTGACAACA	ATTTCCGCTCCTGCTTCTCTG
**COL1A1**	GCTAACCCCCTCCCCAGCCA	GAGCAGGAGCCGGAGGTCCA
**COX4I1**	TGTCCAGCATCCTCTTGGTCT	TCGGTTTCACCGCGCTCGTTA
**GLUT-1 (SLC2A1)**	ATACTCATGACCATCGCGCTAG	AAAGAAGGCCACAAAGCCAAAG
**GPx1**	ACCACGGTCCGGGACTAC	CCGGACGTACTTGAGGGAAT
**HK2**	GCGGCTCAAGACAAGGGGCA	GGCCACCACAGTGCACACCT
**MMP1**	ACAGCTTCCCAGCGACTCTA	CAGGGTTTCAGCATCTGGTT
**MT-ND1**	TCAAACTACGCCCTGATCGG	CAAGGGTCATGATGGCAGGA
**NAMPT**	TCCAATCACAGTGGCCACAA	TGTGTTCTGCTGCTGGAACA
**NNMT**	TCCCCAGTGGTGACCTATGT	TAGTAGCTGCTCTTGAGCGC
**NRF1**	GGAGCAGCTTGCCTCCTCAGA	CCATCACACACATGGGGAGAGCT
**NRF2**	TCAGAAACCAGTGGATCTGCC	GTGACTGAAACGTAGCCGAA
**PDK1**	GAGAGCTTTGGGGTGGATCC	TCACACAGACGCCTAGCATT
**PPIA**	GCCGAGGAAAACCGTGTACTAT	TCTTTGGGACCTTGTCTGCAA
**RPLP0**	CGTCCTCGTGGAAGTGACAT	TAGTTGGACTTCCAGGTCGC
**SDHA**	GCCAGGACCTAGAGTTTGTTC	GCCTTGACTGTTAATGAGAATGC
**SIRT1**	CCAAGGCCACGGATAGGTCCA	ACAGACACCCCAGCTCCAGTT
**SOD1**	GGTGTGGCCGATGTGTCTAT	CCTTTGCCCAAGTCATCTGC
**SOD2**	CTG GAC AAA CCT CAG CCC TAA C	AAC CTG AGC CTT GGA CAC CAA C
**TIMP1**	GGGCTTCACCAAGACCTACA	TGCAGGGGATGGATAAACA

Supplementary table 1. List of primers used for qPCR assays.
