# Supplementary material for: Identification of Functional Cellular Markers Related to Human Health, Frailty and Chronological Age
Source: Aging Cell. 2025 Jul 1;24(9):e70153. doi: 10.1111/acel.70153 (PMC12419852; doi:10.1111/acel.70153)
Supplement: Supplementary file 3 — Table S2. List of the 31 measured cellular parameters used to determine Mahalanobis distance. [file ACEL-24-e70153-s007.pdf]

	Cellular parameters measured
**SENESCENCE**	Number of γH2AX spots per cell<br>Number of p16 spots per cell<br>Nuclear area<br>Cell size<br>Cell granularity<br>β-galactosidase activity<br>Number of γH2AX spots per cell (doxorubicin)<br>Number of p16 spots per cell (doxorubicin)<br>Nuclear area (doxorubicin)<br>Cell size (doxorubicin)<br>Cell granularity (doxorubicin)<br>β-galactosidase activity (doxorubicin)
**STROMA / STRUCTURE**	% CFU-F<br>Extracellular periostin (POSTN)<br>Spontaneous cell migration<br>Cell migration (chemoattractant)
**INFLAMMATION**	Extracellular IL1-β<br>Extracellular IL-6<br>Extracellular IL-10<br>Extracellular TGF-β<br>Extracellular IFN-β<br>Extracellular IL1-β (LPS)<br>Extracellular IL-6 (LPS)<br>Extracellular IL-10 (LPS)<br>Extracellular TGF-β (LPS)<br>Extracellular IFN-β (Poly I:C)
**METABOLISM**	OCR-Basal respiration<br>OCR-Uncoupled respiration<br>ECAR<br>% differentiated cells (adipocytes)<br>Intensity of adipose differentiation

Supplementary table 2. List of the 31 measured cellular parameters used to determine Mahalanobis distance .
