# Supplementary material for: Identification of Functional Cellular Markers Related to Human Health, Frailty and Chronological Age
Source: Aging Cell. 2025 Jul 1;24(9):e70153. doi: 10.1111/acel.70153 (PMC12419852; doi:10.1111/acel.70153)
Supplement: Supplementary file 4 — Table S3. Association of the 60 measured cellular parameters with chronological age or Intrinsic capacity using linear regression and with frailty using logistic regression. Statistical models were applied to assess the relationship between cellular features and clinical variables. Linear regression models were used to evaluate associations between each of the 60 cellular parameters and chronological age or intrinsic capacity (IC), while logistic regression models were used for frailty. For each parameter, the regression coefficient (β), 95% confidence interval (95% CI), and p‐value are reported. r is the Pearson correlation coefficient. Cellular parameters are categorized into four functional groups: senescence, stroma/structure, metabolism and inflammation. Statistically significant associations (p < 0.05) are indicated in bold. [file ACEL-24-e70153-s005.pdf]

		AGE				FRAILTY			IC		
	Cell parameter	β	95% CI	r pearson	p-value	β	95% CI	p-value	β	95% CI	p-value
	Cell doubling time	0.0082	0.005, 0.011	**0.41**	**7.893E-07**	-0.2965	-1.312, 0.719	5,67E-01	1.4152	-1.600, 4.430	3.55E-01
**Stroma Structure**	% CFU-F	-0.006	-0.013, 0.000	-0.16	6.87E-02	0.5463	0.013 , 1.080	**4,50E-02**	-0.6983	-2.205, 0.808	3.61E-01
	ACTA2 mRNA	-0.0017	-0.008, 0.005	-0.07	5.87E-01	-0.1362	-1.226, 0.954	8,07E-01	-1.9790	-5.527, 1.569	2.69E-01
	ACTA2 mRNA fold increase (TGF-β)	0.0003	-0.008, 0.008	0.01	9.31E-01	0.6222	-0.294 ,1.539	1,83E-01	0.5815	-2.155, 3.318	6.72E-01
	CALD1 mRNA	0.0048	-0.014, 0.023	0.07	6.06E-01	0.2363	-0.298 ,0.771	3,86E-01	-0.4440	-1.731, 0.843	4.91E-01
	CALD1 mRNA fold increase (TGF-β)	-0.0007	-0.023, 0.022	-0.01	9.54E-01	-0.2525	-0.687 ,0.182	2,55E-01	0.6418	-0.407, 1.691	2.25E-01
	Cell migration (chemoattractant)	0.0002	-0.006, 0.006	0.01	9.37E-01	0.1534	-0.384, 0.691	5,76E-01	-0.1737	-1.695, 1.347	8.22E-01
	CNN1 mRNA	-0.0055	-0.019, 0.008	-0.11	4.29E-01	0.1504	-0.406, 0.707	5,96E-01	-1.3915	-3.065, 0.282	1.01E-01
	CNN1 mRNA fold increase (TGF-β)	-0.0177	-0.041, 0.006	-0.20	1.34E-01	-0.3096	-0.673, 0.053	9,50E-02	0.0372	-0.056, 0.130	4.25E-01
	COL1A1 mRNA	-0.0034	-0.007, 0.001	-0.21	9.33E-02	0.6475	-1.164, 2.459	4,84E-01	-1.5354	-6.999, 3.928	5.76E-01
	COL1A1 mRNA fold increase (TGF-β)	0.0011	-0.004, 0.006	0.06	6.56E-01	-0.5816	-1.940, 0.776	4,01E-01	2.5944	-1.639, 6.828	2.25E-01
	Extracellular periostin	-0.020	-0.034, -0.007	**-0.27**	**2.60E-03**	-0.4778	-0.792, -0.164	**3,00E-03**	0.9636	0.214, 1.713	**1.20E-02**
	MMP1 mRNA	0.0119	-0.005, 0.029	0.18	1.72E-01	-0.1971	-0.620, 0.226	3,61E-01	-0.9994	-2.255, 0.256	1.16E-01
	MMP1 mRNA fold increase (TGF-β)	-0.0081	-0.025, 0.009	-0.13	3.35E-01	-0.1941	-0.629, 0.241	3,82E-01	0.2007	-0.755, 1.156	6.76E-01
	Spontaneous cell migration	0.0040	-0.003, 0.011	0.10	2.61E-01	-0.0550	-0.536, 0.426	8,23E-01	0.0475	-1.319, 1.413	9.45E-01
	TIMP1 mRNA	0.0097	0.006, 0.013	**0.58**	**7.31E-07**	-1.4714	-3.524, 0.581	1,60E-01	1.8538	-4.250, 7.958	5.46E-01
	TIMP1 mRNA fold increase (TGF-β)	-0.0005	-0.005, 0.004	-0.03	8.27E-01	-1.0881	-2.596, 0.420	1,57E-01	2.9465	-1.546, 7.439	1.94E-01
**Metabolism**	% differentiated cells (adipocytes)	-0.0286	-0.045, -0.012	**-0.29**	**8.06E-04**	-0.0557	-0.250, 0.138	5,74E-01	6.4042	-4.885, 17.694	2,64E-01
	CD36 mRNA	0.0082	-0.002, 0.018	0.21	1.05E-01	0.7690	0.010, 1.528	**4,70E-02**	-0.8981	-3.113, 1.317	4.20E-01
	COX4i1 mRNA	0.0018	-0.000, 0.004	0.23	6.67E-02	-1.9701	-5.813, 1.872	3,15E-01	5.6245	-5.283, 16.532	3.06E-01
	ECAR	0.0033	-0.001, 0.007	0.14	1.11E-01	-0.3142	-1.133, 0.505	4,52E-01	0.3710	-2.019, 2.761	7.59E-01
	GLUT-1 mRNA	0.0044	-0.001, 0.010	0.20	1.17E-01	-1.2756	-2.611, 0.060	6,10E-02	0.2455	-3.839, 4.330	9.05E-01
	GPX1 mRNA	0.0050	0.002, 0.008	**0.44**	**4.03E-04**	-0.3984	-3.024, 2.227	7,66E-01	-7.0998	-15.083, 0.884	8.00E-02
	Hexokinase 2 mRNA	0.0092	0.004, 0.015	**0.40**	**1.44E-03**	-0.0153	-1.325, 1.294	9,82E-01	0.6393	-3.565, 4.844	7.62E-01
	Intensity of adipose differentiation	-0.0007	-0.003, 0.001	-0.06	5.00E-01	1.0075	-0.554 , 2.569	2,06E-01	-0.6004	-11.380, 10.179	9.12E-01
	MT-ND1 mRNA	0.0002	-0.004, 0.005	0.01	9.15E-01	1.0025	-0.658, 2.663	2,37E-01	-3.0314	-7.668, 1.605	1.96E-01
	NAMPT mRNA	0.0026	-0.003, 0.008	0.12	3.58E-01	0.7949	-0.583, 2.173	2,58E-01	-2.1128	-5.869, 1.643	2.65E-01
	NNMT mRNA	0.0017	-0.004, 0.007	0.08	5.48E-01	-0.0782	-1.272, 1.115	8,98E-01	-0.2578	-4.050, 3.534	8.92E-01
	NRF1 mRNA	0.0015	-0.001, 0.004	0.16	2.04E-01	-0.3263	-3.270, 2.618	8,28E-01	-3.4469	-12.521, 5.627	4.50E-01
	NRF2 mRNA	0.0016	-0.001, 0.004	0.17	1.95E-01	0.8491	-2.098, 3.796	5,72E-01	1.0460	-8.174, 10.266	8.21E-01
	OCR-Basal respiration	0.0004	-0.003, 0.004	0.02	8.23E-01	-1.8453	-3.005, -0.686	**2,00E-03**	-69.5866	-377.421, 238.248	6.55E-01
	OCR-Maximal respiration	0.0010	-0.001, 0.003	0.07	4.23E-01	-1.8056	-3.454, -0.157	**3,20E-02**	-295.6297	-940.505, 349.245	3.66E-01
	OCR-Uncoupled respiration	0.0056	0.001, 0.010	**0.22**	**1.21E-02**	-0.3012	-1.074, 0.471	4,45E-01	1.1658	-1.027, 3.359	2.95E-01
	PDK1 mRNA	0.0028	-0.002, 0.008	0.14	2.70E-01	0.2827	-1.106, 1.671	6,90E-01	-3.7298	-8.075, 0.615	9.10E-02
	SDHA mRNA	0.0023	-0.000, 0.005	0.21	9.33E-02	-1.1393	-3.721, 1.442	3,87E-01	4.7389	-3.045, 12.523	2.28E-01
	SIRT1 mRNA	0.0020	-0.001, 0.005	0.18	1.66E-01	1.4978	-1.139, 4.135	2,66E-01	-4.4127	-12.119, 3.293	2.56E-01
	SOD1 mRNA	0.0054	0.003, 0.008	**0.55**	**3.28E-06**	-0.7527	-3.880, 2.374	6,37E-01	2.4609	-7.666, 12.588	6.29E-01
	SOD2 mRNA	0.0044	-0.001, 0.010	0.20	1.09E-01	-0.0330	-1.295, 1.230	9,59E-01	0.0769	-3.912, 4.066	9.69E-01
**Inflammation**	Extracellular IFN-β	0.003	-0.002, 0.007	0,12	1.96E-01	-0.1834	-0.992, 0.625	6,57E-01	0.0371	-2.069, 2.143	9.72E-01
	Extracellular IFN-β (Poly I:C)	0.0107	0.000, 0.021	**0,18**	**4.36E-02**	0.1342	-0.188, 0.457	4,15E-01	0.1173	-0.837, 1.072	8.08E-01
	Extracellular IL-10	0.0008	-0.000, 0.002	0,15	9.17E-02	-1.1043	-4.661, 2.453	5,43E-01	7.0888	-1.730, 15.908	1.14E-01
	Extracellular IL-10 (LPS)	-0.0023	-0.006, 0.001	-0,12	1.75E-01	-0.1407	-1.196, 0.915	7,94E-01	-0.6513	-3.504, 2.202	6.52E-01
	Extracellular IL1-β	0.0006	-0.000, 0.002	0,1	2.60E-01	-1.2396	-4.390, 1.911	4,41E-01	4.5269	-4.290, 13.344	3.12E-01
	Extracellular IL1-β (LPS)	0.0039	0.001, 0.007	**0,23**	**6.83E-03**	-0.6741	-1.911, 0.563	2,85E-01	0.6934	-2.632, 4.018	6.81E-01
	Extracellular IL-6	0.0089	-5.45e-05, 0.018	**0.17**	**5.14E-02**	-0.1314	-0.499, 0.236	4,83E-01	0.1561	-0.919, 1.231	7.74E-01
	Extracellular IL-6 (LPS)	0.0212	0.013, 0.029	**0.42**	**6.32E-07**	0.1122	-0.320, 0.545	6,11E-01	0.1359	-1.037, 1.308	8.19E-01
	Extracellular TGF-β	0.0071	-0.007, 0.021	0,09	3.27E-01	-0.0001	-0.229, 0.229	9,99E-01	0,0017	-0.009, 0.012	7.47E-01
	Extracellular TGF-β (LPS)	-0.0107	-0.029, 0.007	-0,1	2.37E-01	0.0451	-0.137, 0.228	6,28E-01	0.2199	-0.348, 0.787	4.45E-01
**Senescence**	Cell granularity	0.0002	-0.001, 0.001	0.03	7.520E-01	-0.2841	-2.843, 2.275	8,28E-01	0.8947	-6.459, 8.249	8.10E-01
	Cell granularity (doxorubicin)	-0.0003	-0.002, 0.001	-0.04	6.425E-01	-0.0959	-2.536, 2.344	9,39E-01	-0.3510	-7.447, 6.745	9.22E-01
	Cell size	-0.0003	-0.001, 0.001	-0.06	5.137E-01	1.0755	-3.085, 5.236	6,12E-01	-5.2764	-17.167, 6.614	3.82E-01
	Cell size (doxorubicin)	0.0011	0.000, 0.002	0.21	1.597E-02	0.7726	-2.802, 4.347	6,72E-01	-2.7934	-13.042, 7.455	5.91E-01
	Nucelus area	0.0010	-0.000, 0.002	0.15	7.641E-02	-1.4977	-4.465, 1.469	3,22E-01	0.2835	-11.941, 12.508	9.63E-01
	Nucleus area (doxorubicin)	-9.024e-06	-0.001, 0.001	-0.00	9.875E-01	-2.1435	-5.220, 0.933	1,72E-01	2.7428	-5.466, 10.951	5.10E-01
	p16 spots per cell	0.0103	0.001, 0.020	**0.19**	**3.212E-02**	-0.2643	-0.617, 0.089	1,42E-01	0.2870	-0.704, 1.278	5.68E-01
	p16 spots per cell (doxorubicin)	0.0021	-0.003, 0.007	0.07	4.175E-01	0.0445	-0.589, 0.678	8,90E-01	-1.5059	-3.335, 0.323	1.06E-01
	γH2AX spots per cell	0.0127	0.007, 0.018	**0.38**	**7.765E-06**	-0.2092	-0.814, 0.396	4,98E-01	0.1567	-1.634, 1.948	8.63E-01
	γH2AX spots per cell (doxorubicin)	-0.0025	-0.007, 0.002	-0.10	2.774E-01	-0.4957	-1.239, 0.248	1,91E-01	-0.0684	-2.169, 2.032	9.49E-01
	β-galactosidase	-0.0014	-0.005, 0.002	-0.07	4.43E-01	0.2091	-0.743, 1.161	6,67E-01	-8.594e-05	-0.000, 0.000	6.75E-01
	β-galactosidase (doxorubicin)	0.0034	0.000, 0.006	**0.19**	**3.16E-02**	0.3415	-0.760, 1.443	5,43E-01	0.2475	-2.809, 3.304	8.73E-01

Supplementary Table 3. Association of the 60 measured cellular parameters with chronological age or Intrinsic capacity and with frailty.
