# Supplementary figures and images for: Identification of Functional Cellular Markers Related to Human Health, Frailty and Chronological Age
Source: Aging Cell. 2025 Jul 1;24(9):e70153. doi: 10.1111/acel.70153 (PMC12419852; doi:10.1111/acel.70153)

A.

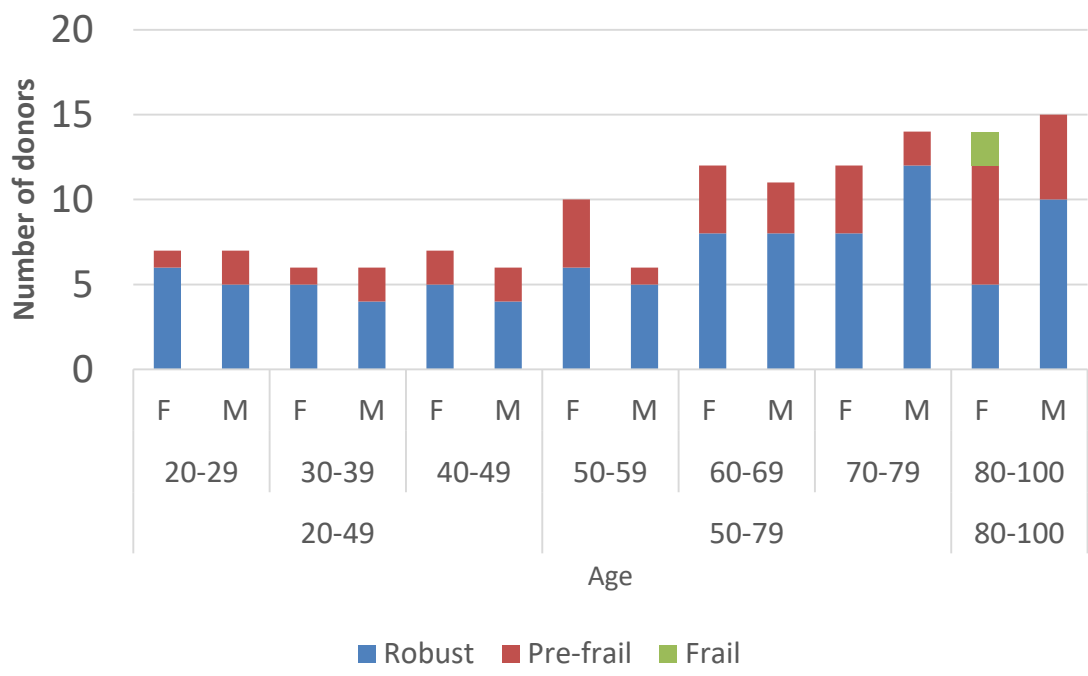

B.

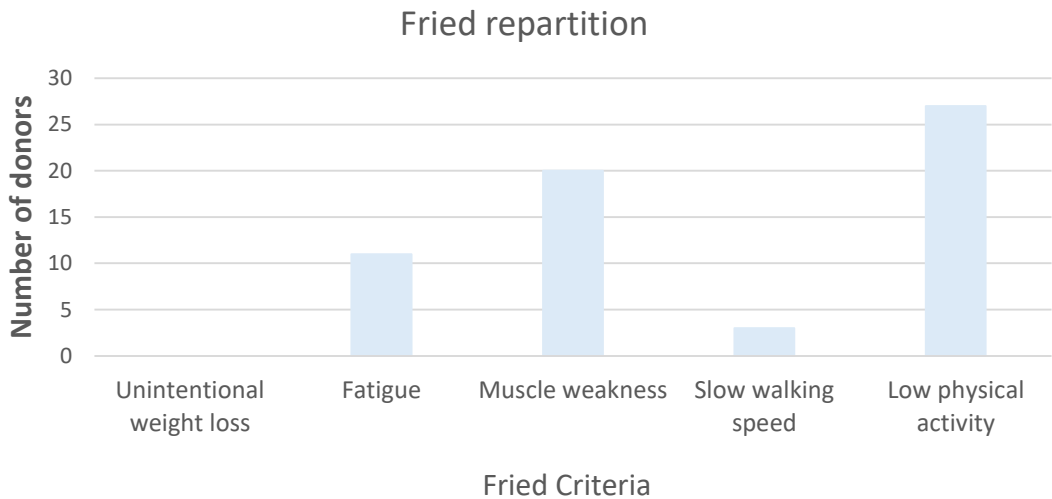

C.

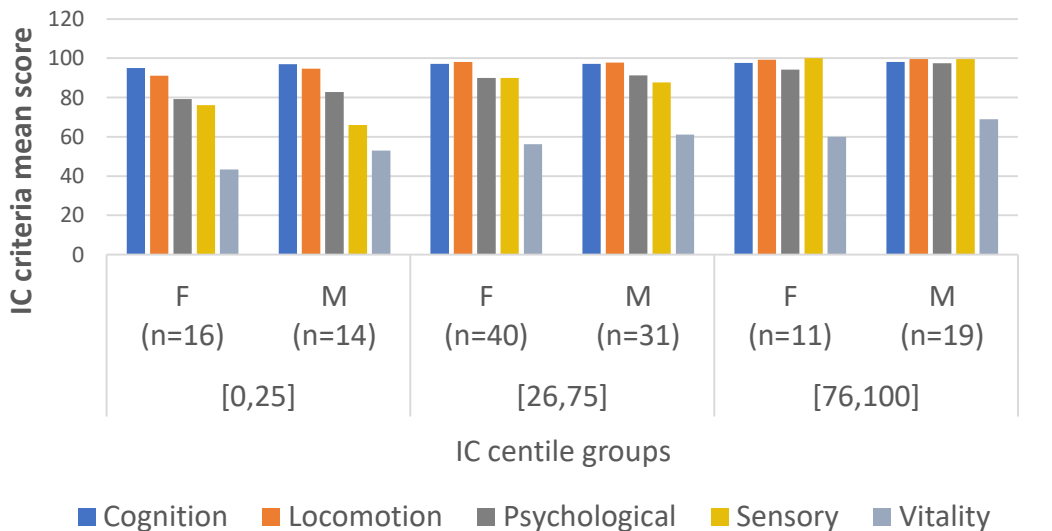

Supplementary figure 1. Study population

Supplement: Supplementary file 5 — Figure S1. Study population. Distribution of individuals in the fibroblast cohort from INSPIRE‐T cohort according to their chronological age range, sex and frailty status (robust, pre‐frail, frail) (A) and to Fried’s criteria (unintentional weight loss, fatigue, weakness, slow walking speed and low physical activity) (B). Distribution of average Intrinsic Capacity domain scores (Cognition, Locomotion, Psychological, Vitality and Sensory) by IC centile groups and sex (male and female), including the number of individuals in each group (C). [file ACEL-24-e70153-s004.pdf]

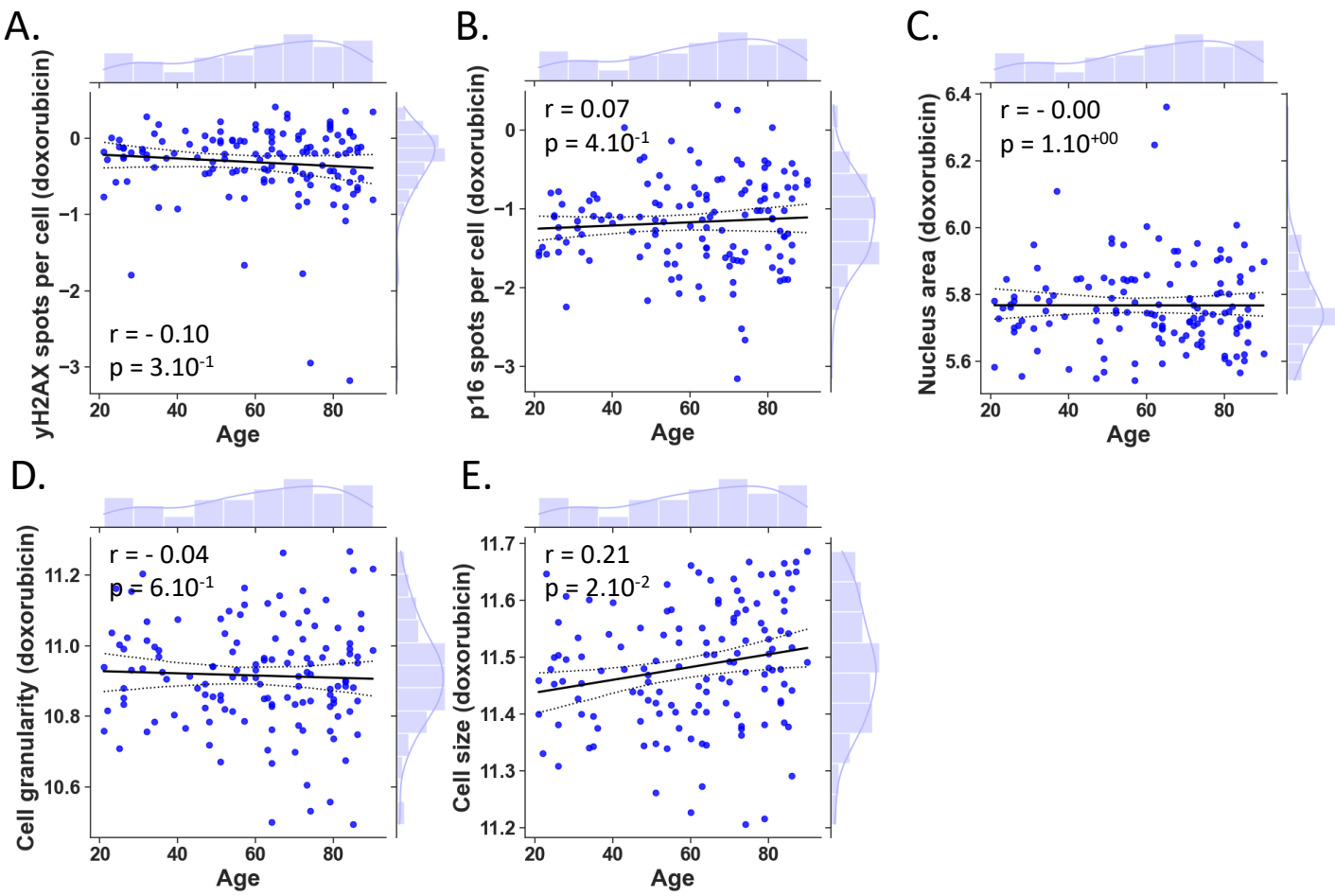

**Supplementary figure 3. Fibroblasts response to doxorubicin exposure with chronological age.**

Supplement: Supplementary file 7 — Figure S3. Fibroblast response to doxorubicin exposure with chronological age. Linear regression with marginal distribution represents cell parameters as a function of age. Association of number of γH2AX foci per cell (A), number of p16 spots per cell (B), nuclear area (μm2) (C) and cell granularity (a.u) (D) and cell size (E) with age after doxorubicin challenge are shown. The black line represents the regression line and the dashed line show the 95% confidence of the fit. Histograms depict the marginal distribution of the respective variable. r and p‐value represent the Pearson correlation coefficient, and the associated p‐value for each measured parameter with age. A p‐value < 0.05 was considered significant (A–E). [file ACEL-24-e70153-s008.pdf]
