# Supplementary material for: Identification of Functional Cellular Markers Related to Human Health, Frailty and Chronological Age
Source: Aging Cell. 2025 Jul 1;24(9):e70153. doi: 10.1111/acel.70153 (PMC12419852; doi:10.1111/acel.70153)
Supplement: Supplementary file 6 — Figure S2. Stroma and structure characteristics of skin fibroblasts with chronological age. Linear regression with marginal distribution represents cell parameters as a function of chronological age. Correlation between age and nuclear area (μm2) (A), cell size (a.u) (B), cell granularity (a.u) (C), β‐galactosidase (doxorubicin) (MFI) (D), fibroblast clonogenicity potential (% CFU‐f) (E), spontaneous cell migration (a.u) (F) and cell migration in response to 10% FBS as chemoattractant (a.u) (G) are shown. Association of COL1A1 and MMP1 mRNA expression (2−ΔCt) with age are shown (H–I). The black line represents the regression line and the dashed line show the 95% confidence of the fit. Histograms depict the marginal distribution of the respective variable. r and p‐value represent the Pearson correlation coefficient, and the associated p‐value for each measured parameter with age. A p‐value < 0.05 was considered significant (A–I). [file ACEL-24-e70153-s011.pdf]

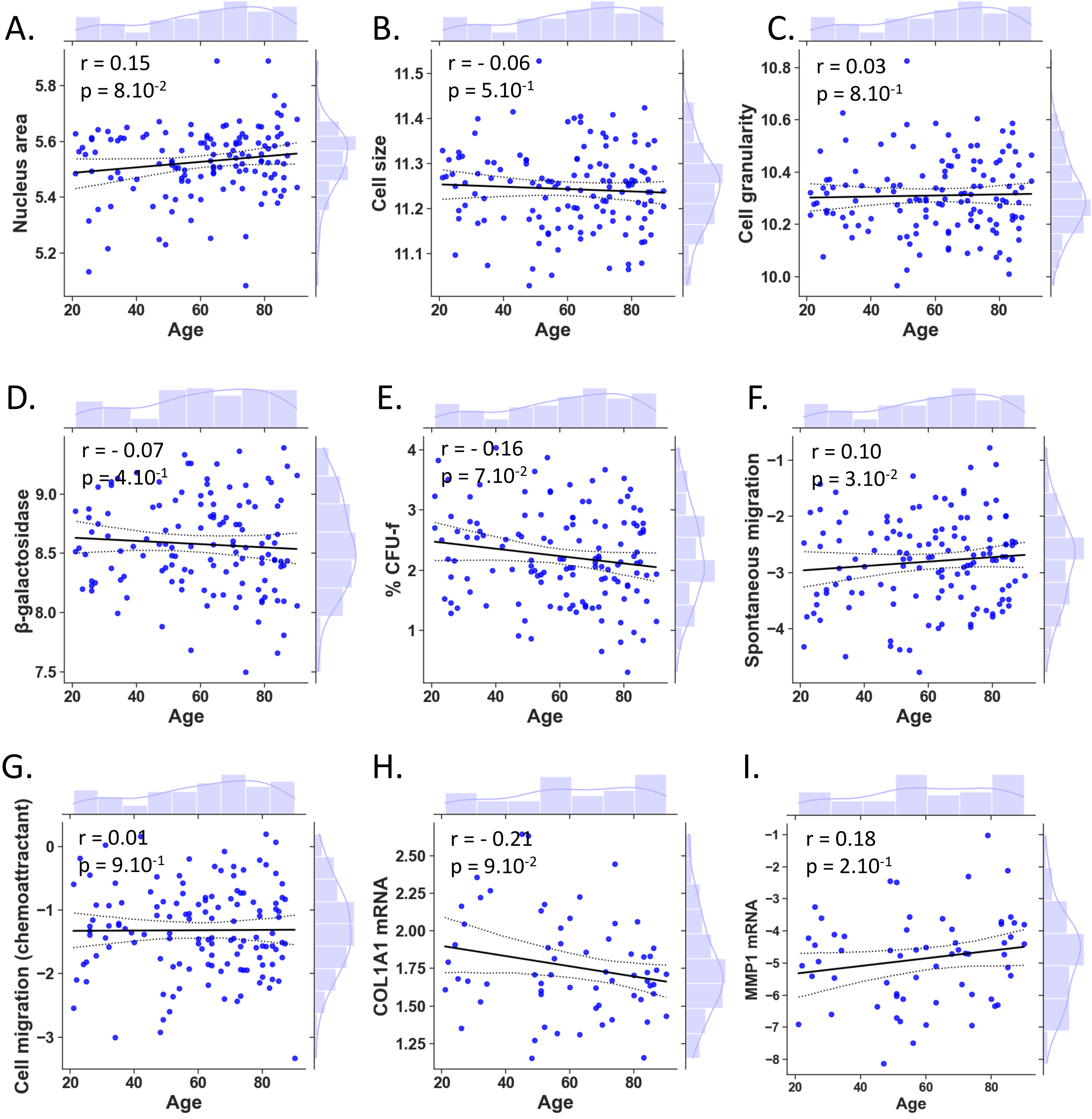

Supplementary figure 2. Stroma and Structure characteristics of skin fibroblasts with chronological age.
