# Supplementary material for: Identification of Functional Cellular Markers Related to Human Health, Frailty and Chronological Age
Source: Aging Cell. 2025 Jul 1;24(9):e70153. doi: 10.1111/acel.70153 (PMC12419852; doi:10.1111/acel.70153)
Supplement: Supplementary file 8 — Figure S4. Fibroblast ability to differentiate into myofibroblasts and adipocytes with chronological age. Linear regression with marginal distribution represents cell parameters as a function of age. Association of ACTA2 (A), CALD1 (B), CNN1 (C) mRNA expression fold increase to control cells (2−ΔΔCt) with age after myofibroblastic differentiation (2 ng/mL of TGF‐β1 for 10 days) are shown. Correlation between age and intensity of adipose differentiation (D) is shown after 14 days of adipose differentiation induction. The black line represents the regression line and the dashed line show the 95% confidence of the fit. Histograms depict the marginal distribution of the respective variable. r and p‐value represent the Pearson correlation coefficient, and the associated p‐value for each measured parameter with age. A p‐value < 0.05 was considered significant (A–D). [file ACEL-24-e70153-s010.pdf]

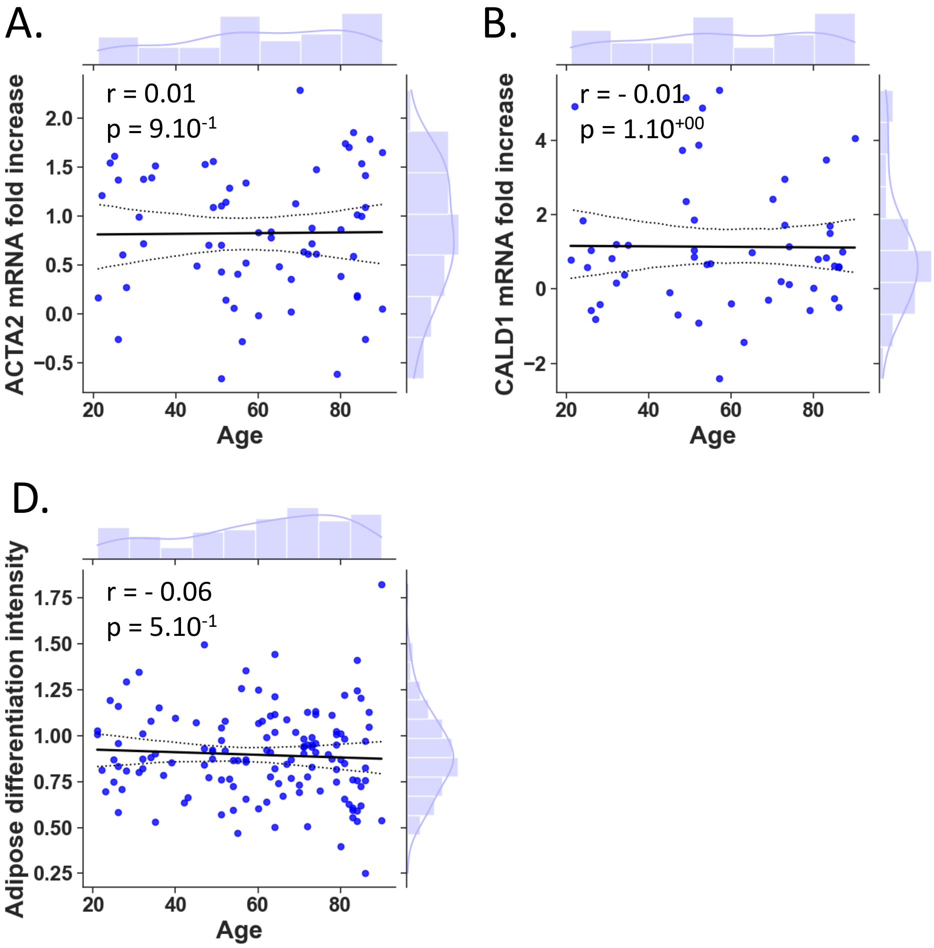


**Supplementary figure 4. Fibroblasts ability to differentiate into myofibroblasts and adipocytes with chronological age.**
