# Supplementary material for: Identification of Functional Cellular Markers Related to Human Health, Frailty and Chronological Age
Source: Aging Cell. 2025 Jul 1;24(9):e70153. doi: 10.1111/acel.70153 (PMC12419852; doi:10.1111/acel.70153)
Supplement: Supplementary file 9 — Figure S5. Cytokine production by fibroblasts under basal conditions and in response to inflammatory stress with aging. Linear regression with marginal distribution represents cell parameters as a function of age. Correlation between age with extracellular IL1‐β (A), IL‐10 (B), TGF‐β (C), and IFN‐β (D) concentration (pg/mL/105 cells) are shown. Association of extracellular IL‐10 (E) and TGF‐β (F) concentration (pg/mL/105 cells) with age after LPS stimulation (1 μg/mL) are shown. The black line represents the regression line and the dashed line show the 95% confidence of the fit. Histograms depict the marginal distribution of the respective variable. r and p‐value represent the Pearson correlation coefficient, and the associated p‐value for each measured parameter with age. A p‐value < 0.05 was considered significant (A–F). [file ACEL-24-e70153-s006.pdf]

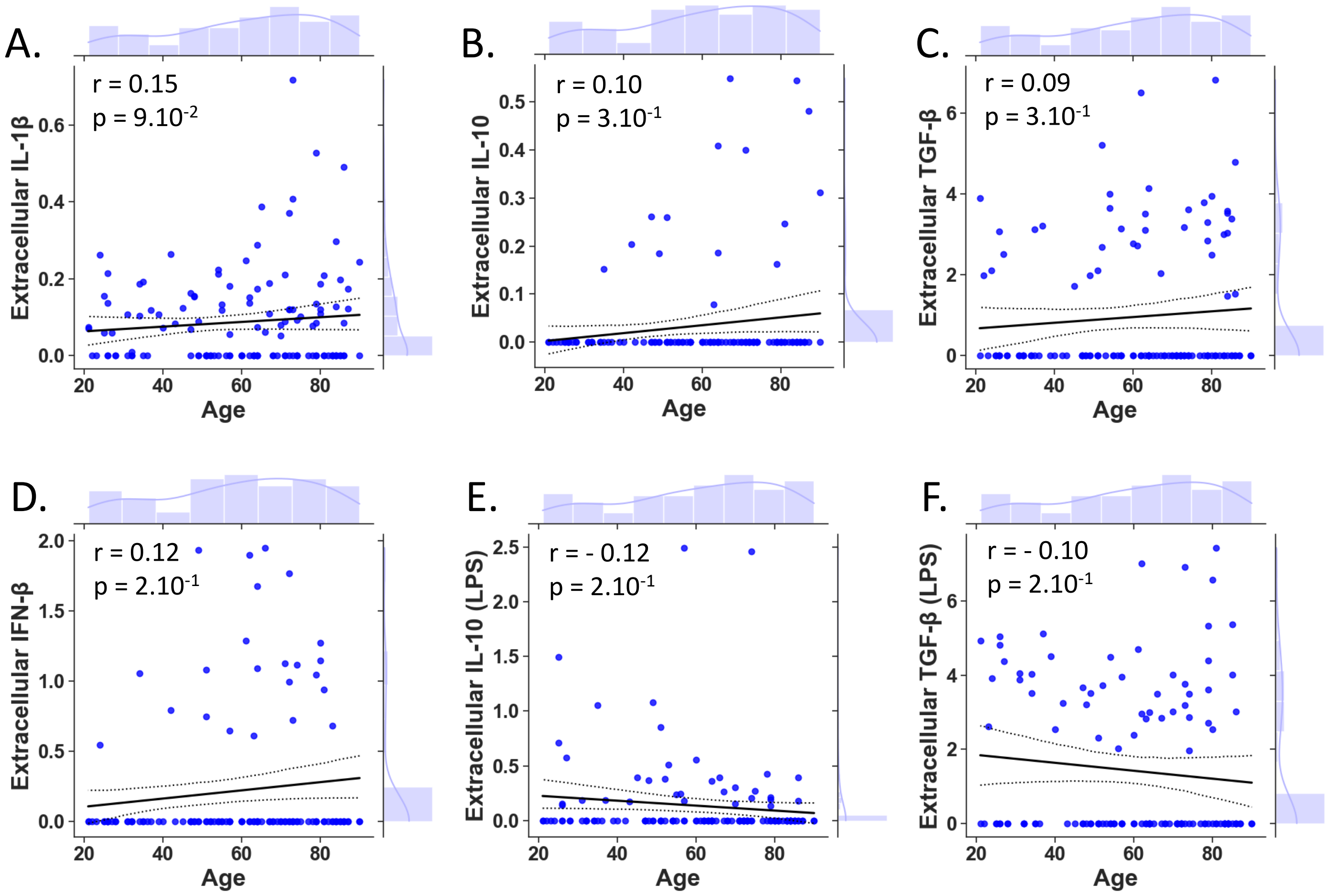

Supplementary figure 5. Cytokine production by fibroblasts under basal conditions and in response to inflammatory stress with ageing.
