# Supplementary material for: Identification of Functional Cellular Markers Related to Human Health, Frailty and Chronological Age
Source: Aging Cell. 2025 Jul 1;24(9):e70153. doi: 10.1111/acel.70153 (PMC12419852; doi:10.1111/acel.70153)
Supplement: Supplementary file 10 — Figure S6. Chronological age‐related variation in metabolic abilities and in the expression of genes regulating metabolism. Linear regression with marginal distribution represents cell parameters as a function of age. Correlation between age with basal (A) and maximal (B) mitochondrial respiration (pmol/min/2.104 cells), ECAR (C) and GLUT‐1 (D), NRF1 (E), SDHA (F), COX4i1 (G), MT‐ND1 (H), PDK1 (I), SIRT1 (J), NRF2 (K) and SOD2 (L) mRNA expression (2−ΔCt) are shown. The black line represents the regression line and the dashed line show the 95% confidence of the fit. Histograms depict the marginal distribution of the respective variable. r and p‐value represent the Pearson correlation coefficient, and the associated p‐value for each measured parameter with age. A p‐value < 0.05 was considered significant (A–L). [file ACEL-24-e70153-s009.pdf]

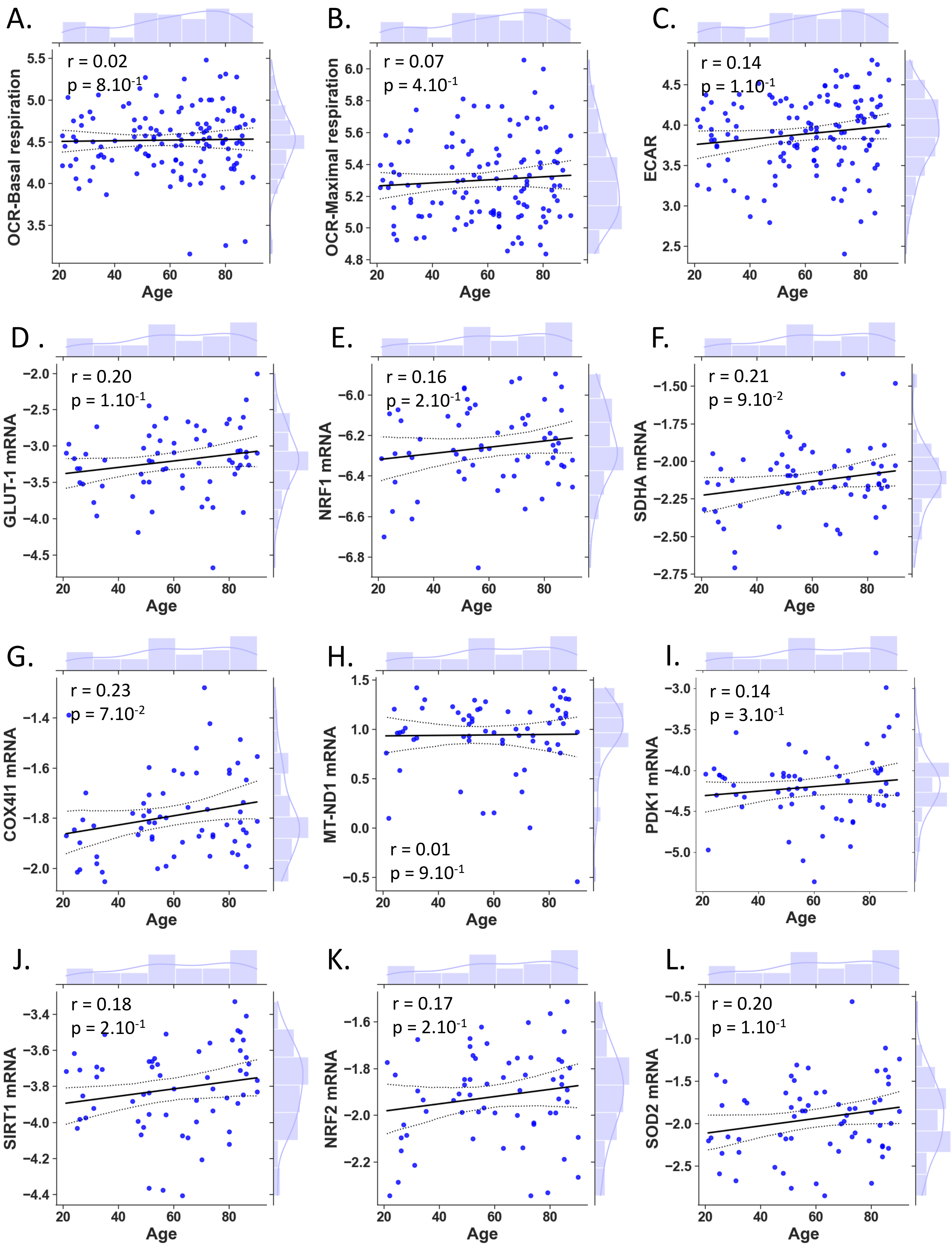

Supplementary figure 6. Chronological age-related variation in metabolic abilities and in the expression of genes regulating metabolism.
