# Supplementary material for: Identification of Functional Cellular Markers Related to Human Health, Frailty and Chronological Age
Source: Aging Cell. 2025 Jul 1;24(9):e70153. doi: 10.1111/acel.70153 (PMC12419852; doi:10.1111/acel.70153)
Supplement: Supplementary file 11 — Figure S7. Correlation matrix for variables significantly correlated with chronological age. Pearson’s correlation coefficients were calculated to indicate the strength of the relationship between two variables. The color scale represents the value of the correlation. A p‐value < 0.05 was considered significant (*p‐value < 0.05, **p‐value < 0.01, ***p ‐value < 0.001 and ****p ‐value < 0.0001), and those > 0.05 were considered nonsignificant (ns). Correlation matrix of 11 parameters measured on fibroblasts from 119 donors (A). Correlation matrix of 15 parameters measured on fibroblasts from 57 donors (B). [file ACEL-24-e70153-s002.pdf]

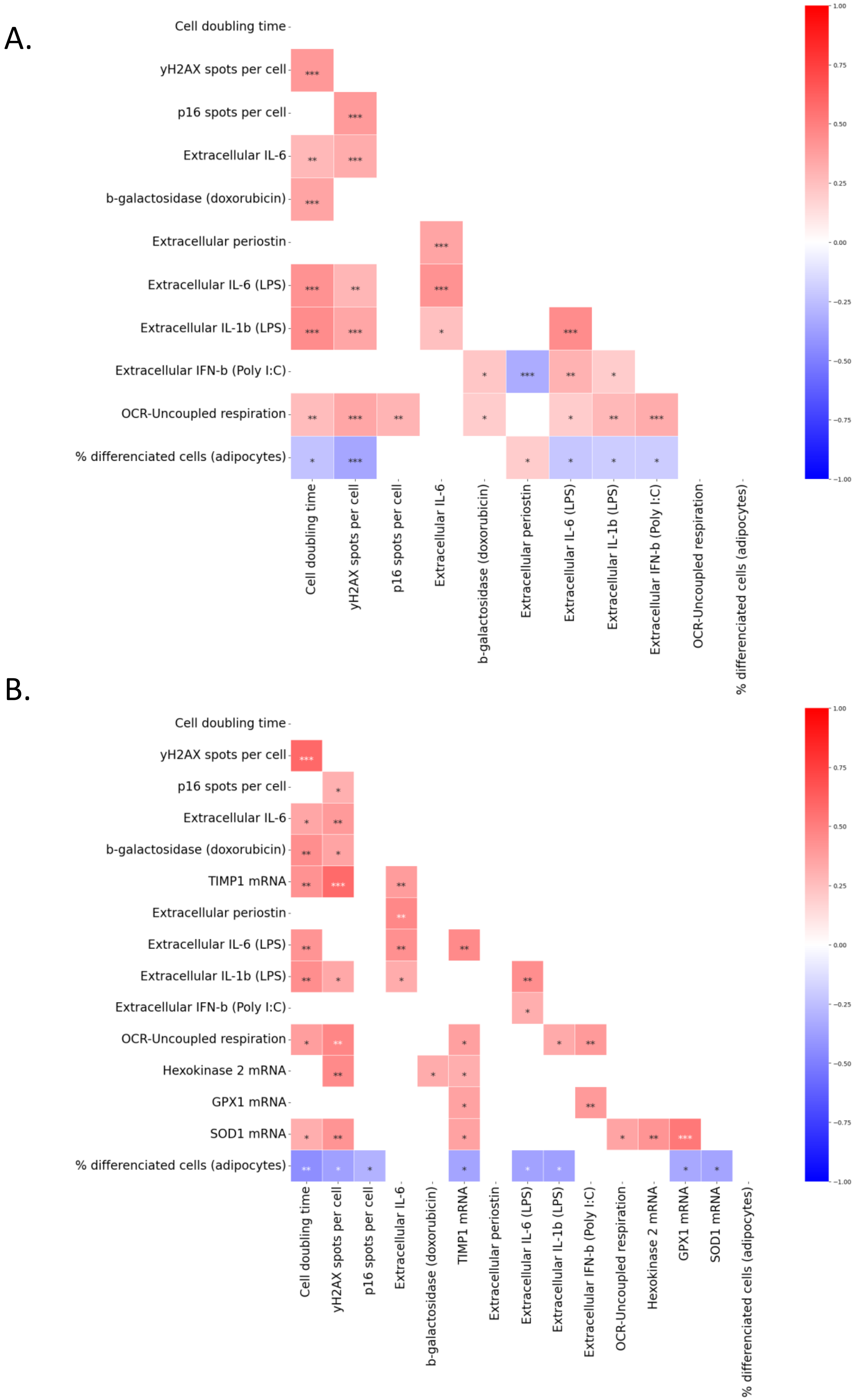

Supplementary figure 7. Correlation matrix for variables significantly correlated with chronological age.
